# Detergent Screening and Purification of the Human Liver ABC Transporters BSEP (ABCB11) and MDR3 (ABCB4) Expressed in the Yeast *Pichia pastoris*

**DOI:** 10.1371/journal.pone.0060620

**Published:** 2013-04-04

**Authors:** Philipp Ellinger, Marianne Kluth, Jan Stindt, Sander H. J. Smits, Lutz Schmitt

**Affiliations:** Institute of Biochemistry, Heinrich Heine University, Düsseldorf, Germany; University of Technology Sydney, Australia

## Abstract

The human liver ATP-binding cassette (ABC) transporters bile salt export pump (BSEP/ABCB11) and the multidrug resistance protein 3 (MDR3/ABCB4) fulfill the translocation of bile salts and phosphatidylcholine across the apical membrane of hepatocytes. In concert with ABCG5/G8, these two transporters are responsible for the formation of bile and mutations within these transporters can lead to severe hereditary diseases. In this study, we report the heterologous overexpression and purification of human BSEP and MDR3 as well as the expression of the corresponding C-terminal GFP-fusion proteins in the yeast *Pichia pastoris*. Confocal laser scanning microscopy revealed that BSEP-GFP and MDR3-GFP are localized in the plasma membrane of *P. pastoris*. Furthermore, we demonstrate the first purification of human BSEP and MDR3 yielding ∼1 mg and ∼6 mg per 100 g of wet cell weight, respectively. By screening over 100 detergents using a dot blot technique, we found that only zwitterionic, lipid-like detergents such as Fos-cholines or Cyclofos were able to extract both transporters in sufficient amounts for subsequent functional analysis. For MDR3, fluorescence-detection size exclusion chromatography (FSEC) screens revealed that increasing the acyl chain length of Fos-Cholines improved monodispersity. BSEP purified in n-dodecyl-β-D-maltoside or Cymal-5 after solubilization with Fos-choline 16 from *P. pastoris* membranes showed binding to ATP-agarose. Furthermore, detergent-solubilized and purified MDR3 showed a substrate-inducible ATPase activity upon addition of phosphatidylcholine lipids. These results form the basis for further biochemical analysis of human BSEP and MDR3 to elucidate the function of these clinically relevant ABC transporters.

## Introduction

ATP-binding cassette (ABC) transporters constitute one of the largest families of membrane transport proteins present in all three kingdoms of life. They transport a wide variety of different substrates ranging from small ions to large proteins across biological membranes using ATP as energy source [1], [2]. ABC transporters are composed of two transmembrane domains (TMDs) and two highly conserved nucleotide-binding domains (NBDs). TMDs determine the substrate specificity and the NBDs fuel the transport by binding and hydrolyzing ATP. In eukaryotes, the TMDs and NBDs are encoded on one gene and build up either a full-size transporter (one gene encoding two TMDs and two NBDs) or a half-size transporter (one gene encoding one TMD and one NBD), which hetero- or homodimerize to form the functional unit.

Within the human genome 48 genes encode for ABC proteins, which are involved mainly in transport [3]. Mutations in these ABC protein genes can lead to severe diseases such as cystic fibrosis, X-linked Adrenoleukodystrophy or Tangier disease. Beside this, ABC transporters are also involved in processes like multidrug resistance of cancer cells [4], [5], [6], [7]. In hepatocytes, eleven ABC transporters are expressed. Except for the transport of different cyclic nucleotides, glucuronide and glutathione conjugates through MRPs (MRP 1–6, note that MRP1 is detected only in fetal hepatocytes) [8] and the transport of endo- and xenobiotics by MDR1 (P-gp) [9] and ABCG2 [10], one of the main function of ABC transporters in the liver is the formation of bile depending on the ABC transporters BSEP (ABCB11), MDR3 (ABCB4) and ABCG5/8 [11]. Bile is essential for the digestion of fat as well as for the absorption of lipids and fat-soluble vitamins originating from food ingestion in the small intestine. In the intestine the main components of bile, bile salts and phosphatidylcholine are recycled via the enterohepatic circulation [12]. Bile salts, phosphatidylcholine and cholesterol form mixed micelles in the canaliculus, which dampen the detergent effect of the amphiphatic bile salts as well as prevent the formation of cholesterol crystals. Bile formation is dependent on the three ABC transporters BSEP (ABCB11), MDR3 (ABCB4) and ABCG5/8 [13].

The bile salt export pump (BSEP) is the main bile salt transporter in humans and is localized in the apical membrane of hepatocytes [14]. It is a 1321 amino acid large, glycosylated full-size ABC transporter and mediates the ATP-dependent bile flow by transporting monovalent bile salts like taurine and glycine conjugates of primary and secondary bile salts (e.g. tauro- and glycocholate or taurodeoxycholate) into the canaliculus [15]. The human multidrug resistance protein 3 (MDR3) is a close homologue of MDR1 (P-glycoprotein, ABCB1) with an amino acid sequence identity of nearly 80%. However, MDR3 exclusively translocates phosphatidylcholine from the inner to the outer leaflet of the apical membrane [16]. MDR3 is like BSEP a glycosylated full-size transporter composed of 1288 amino acid [17]. The heterodimeric ABC transporter ABCG5/G8 completes the bile forming machinery by transporting cholesterol [18], [19].

Mutations within the *BSEP* and *MDR3* gene can lead to different cholestatic diseases, e.g. progressive familiar intrahepatic cholestasis type 2 and 3 (PFIC2 and PFIC3) [20], [21], [22], benign recurrent intrahepatic cholestasis type 2 (BRIC2) [23] or intrahepatic cholestasis of pregnancy (ICP) [24] and low-phospholipid associated cholestasis (LPAC) [25]. Therapy for cholestatic disease includes treatment with e.g. ursodeoxycholic acid or surgical biliary diversion [26]. If none of those treatments is successful, the only alternative therapy is liver transplantation. New successful forms of therapy include treatment with chemical chaperones like 4-phenylbutyrate for misfolded BSEP mutants [27].

Because of their high clinical interest, MDR3 and especially BSEP have been characterized extensively in cell culture as well as animal models [22], [28], [29], [30], [31], [32]. A well-established system for investigating BSEP are for example insect cell-based vesicles, which allow to perform transport studies and to study kinetics, inhibitors or mutants [13], [33]. Less is known about MDR3, because of the difficulty to establish a robust activity assay. Together, all these assays are performed in whole cells or membranes and not with the isolated proteins.

To investigate the function of BSEP and MDR3 in its isolated form, a substantial expression of these proteins is required. To date, no reports regarding the purification of both proteins from cell culture systems or other expression systems have been reported. An alternative to cell culture is the use of yeast expression systems such as *Saccharomyces cerevisiae* or *Pichia pastoris*, which also harbor the eukaryotic protein processing machinery and can be grown to high cell densities. Chloupková *et al*. tested 25 human ABC transporters for expression in *P. pastoris* [34], but BSEP and MDR3 were not included in this study, while for example MRP2, another human liver ABC transporter, could not be expressed.

In general, *S. cerevisiae* has been used frequently to express eukaryotic membrane proteins [35]. After successful establishment of an expression system, the purification of a membrane protein requires first of all its solubilization with detergents from the membrane of the expression host. However, finding an adequate detergent for extraction and purification that preserves the membrane protein in a stable and functional form is an empirical process. High throughput methods have been developed in order to screen the influence of detergents on stability and monodispersity of the purified membrane protein [36], [37], [38], [39], [40], [41], [42]. One of these approaches is fluorescence-detection size exclusion chromatography (FSEC) based on the fluorescence of a green fluorescent protein (GFP) tag fused to the membrane protein. In this approach solubilized crude membranes are loaded on a size exclusion column and the elution is monitored via the fluorescence of GFP. Thereby only the membrane-GFP fusion protein is visible and the result can be evaluated based on the shape of the elution peak [43], [44]

In this study, we established the heterologous overexpression in the yeast *P. pastoris* and the subsequent solubilization and purification of human BSEP and MDR3. To achieve this, we applied a dot blot technique and FSEC to identify the most suitable detergent for BSEP and MDR3. The purified protein could be isolated in a functional state as judged by substrate-induced ATPase activity of MDR3 and ATP binding in the case of BSEP.

## Materials and Methods

### Materials

All detergents were obtained from Affymetrix with the exception of Digitonin, which was purchased from Sigma. Lipids were from Sigma or Avanti Polar Lipids.

### Routine Procedures

SDS-PAGE on 7% gels used the Bio-Rad Minigel system. Immunoblotting followed standard procedures using the monoclonal anti-P-gp C219 antibody in case of MDR3 (Abcam), the F-6 anti-BSEP antibody (Santa Cruz Biotechnology) or an anti-GFP antibody (Sigma). Protein concentration was estimated by the Bradford method using a Coomassie Plus Assay (Pierce).

### Cloning of human BSEP and MDR3 and GFP fusion expression constructs for *Pichia pastoris*

The general cloning procedure is described in detail in Stindt *et al.* [45]. The *P. pastoris* expression vector pSGP18 was made compatible for *Saccharomyces cerevisiae* by introducing a 2µ origin of replication into its backbone. The 2µ origin of replication was PCR-amplified from the YEpHIS vector with the primer pairs 2µ for pPIC S1 and 2µ for pPIC S2 (for oligonucleotide sequences see Table 1). The resulting PCR product and the pSGP18 vector were digested with *Pci*I and ligated yielding pSGP18-2µ. The coding sequences for human BSEP and MDR3 (NCBI accession code: NM_003742.2 and NM_000443.3) were PCR-amplified with the primer pairs BSEP-HR-PP-S1 and BSEP-HR-PP-S2 and MDR3-HR-PP-S1 and MDR3-HR-PP-S2, respectively. For *Pichia* expression of the GFP-tagged transporters, the respective coding sequences were amplified either with the primer pair BSEP-PP-HR-S1 and YEpN14HIS-BSEP-S2 or with MDR3-PP-HR-S1 and YEpN14HIS-MDR3-S2. The S65T-GFP sequence of pFA6a-GFP(S65T)-kanMX6 [27] was either amplified with primer pair GFP-BSEP-HR-S1 and GFP-PP-HR-S2 or primer pair GFP-MDR3-HR-S1 and GFP-PP-HR-S2. This includes the necessary homologous overlaps to the PCR products for in-frame recombination into pSGP18-2µ. pSGP18 contains a 3C protease cleavage site, a calmodulin binding peptide (CBP) tag and a RGS-6xhis-tag C-terminal to the proteins in the multiple cloning site [34]. For expression of the GFP-fusion proteins, tags were replaced by GFP in the process of recombination. The *Bsm*BI linearized pSGP18-2µ vector and the PCR fragments were gel-purified, mixed in equimolar amounts (either with BSEP or MDR3 or each together with GFP) and transformed into *S. cerevisiae* [45]. The ATP hydrolysis deficient mutant of MDR3 was generated by introduction of two point mutations in the conserved NBD. Therefore, we replaced Glu 558 and Glu 1207 of the Walker B motif to Gln using the QuikChange® XL Site-Directed Mutagenesis Kit (Agilent Technologies). The sequence of all constructs were verified by DNA sequencing.

**Table 1:** PCR oligonucleotides used in this study.

Oligonucleotide	Sequence 5′→ 3′
pSGP18-2µ-ori-S1	TAATACGGTTATCCACAGAATCAGGGGATAACGCAGGAAAGAACATGTAAATATTGCGAATACCGCTTCCACAAACATTG
pSGP18-2µ-ori-S2	AACGCGGCCTTTTTACGGTTCCTGGCCTTTTGCTGGCCTTTTGCTCACATGTTATTTCACACCGCATATATCGGATCGTACT
BSEP-HR-PP-S1	ATCAAAAAACAACTAATTATTCGAACGAGGTAAAAGAATGTCTGACTCAGTAATTCTTCGAAGT ATA
BSEP-HR-PP-S2	ACGTTTGGACCTTGGAAAAGACTTCTAAGGAGTTGGAGGCACTGATGGGGGATCCAGTGGTGACTAGTTT
MDR3-HR-PP-S1	ATCAAAAAACAACTAATTATTCGAACGAGGTAAAAGAATGGATCTTGAGGCGGCAAAGAACGGAACA
MDR3-HR-PP-S2	ACGTTTGGACCTTGGAATAAGACTTCTAAGGAGTTGGAGGCTAAGTTCTGTGTTCCAGCCTGGACACTGACCATTGAAAAATAG
YEpN14HIS-BSEP-S2	GAATAAGGTAAACATGGTAGCGATGTCGACCTCGAGACGCGTCTAACTGATGGGGGATCCAGTGGTGACT
YEpN14HIS-MDR3-S2	GAATAAGGTAAACATGGTAGCGATGTCGACCTCGAGACGCGTCTATAAGTTCTGTGTCCCAGCCTGGACACTGACCATT
GFP-BSEP-HR-S1	AGCCTACTACAAACTAGTCACCACTGGATCCCCCATCAGTGGTGGTGGTCGACGGATCCCCGGGTTA
GFP-PP-HR-S2	ACGTTTGGACCTTGGAATAAGACTTCTAAGGAGTTGGAGGCTATTATTTGTATAGTTCATCCATGCCATGT
GFP-MDR3-HR-S1	TTTCAATGGTCAGTGTCCAGGCTGGAACAAAGAGACAAGGTGGTGGTCGACGGATCCCCGGGTTA
MDR3-E558Q S1	GATCCTTCTGCTGGATCAAGCCACGTCAGCATTGGACAC
MDR3-E558Q S2	GTGTCCAATGCTGACGTGGCTTGATCCAGCAGAAGGATC
MDR3-E1207Q S1	CAAATCCTCCTGTTGGATCAAGCTACATCAGCTCTGGATAC
MDR3-E1207Q S2	GTATCCAGAGCTGATGTAGCTTGATCCAACAGGAGGATTTG

### Transformation of *P. pastoris*

*BSEP* and *MDR3* expression constructs were transformed into competent *P. pastoris* X33 (Invitrogen) cells using standard procedures (Invitrogen). Briefly, 10-20 µg DNA of the expression construct were linearized using *Pme*I (New England Biolabs) to facilitate homologous recombination at the *AOX*1 locus, extracted by phenol/chloroform, re-suspended in 10 µl sterile H_2_O and transformed into 80 µl electro-competent *P. pastoris* cells by electroporation (1.500 V, 5 ms). Cells were incubated in 1 M sorbitol without shaking for 1 h at 30°C, 1 ml YPD was subsequently added and cells were shaken for 2 h at 200 rpm and 30°C. 100 µl of this suspension was plated onto YPDS plates containing 100 µg/ml Zeocin or higher and incubated for 30°C until colonies appeared. 10 to 20 colonies were re-streaked on YPD plates containing Zeocin and used for expression studies.

### Expression screening of *BSEP* and *MDR3* transformed *P. pastoris* cells

Small-scale expression screens of *BSEP* or *MDR3 P. pastoris* clones were performed similarly as described by Wang *et al*. [46]. 50 ml cultures were grown overnight in MGY medium (1.34% (w/v) yeast nitrogen base, 1% (v/v) glycerol and 4×10^−5^% (w/v) biotin) at 30°C and 220 rpm, harvested by centrifugation, re-suspended in 50 ml MMY (1.34% (w/v) yeast nitrogen base, 0.5% (v/v) methanol and 4×10^−5^% (w/v) biotin) and incubated for another 24 h to induce protein expression. 2 ml of these cells were harvested, washed in 2 ml of homogenization buffer (50 mM Tris-HCl, pH 8.0, 0.33 M sucrose, 75 mM NaCl, 1 mM EDTA, 1 mM EGTA, 100 mM 6-Aminocaproic acid, 2 mM β-Mercaptoethanol) supplemented with protein inhibitor cocktail (Roche) and re-suspended in 500 µl of homogenization buffer. Cells were lysed with 1 ml of acid-washed zirconia beads (Roth) by vortexing 6 times for 1 min with 1 min breaks on ice. Disrupted cells were centrifuged for 5 min, 12.000 xg, 4°C and the supernatant was adjusted to 10 mM MgCl_2_ and incubated on ice for 15 min. Precipitated membranes were harvested by centrifugation for 30 min, 20.000 xg, 4°C and the resulting pellet was re-suspended in SDS sample buffer and loaded onto a 7% SDS-PAGE. Expression was visualized by immuno blotting.

### Fermentation of BSEP and MDR3

For large-scale expression, BSEP, BSEP-GFP and MDR3 expressing clones were fermented in a 15 liter table-top glass fermentor (Applikon Biotechnology) according to the Invitrogen *Pichia* fermentation guidelines [26] using the basal salt media. Typically a volume of 6 l media was inoculated with 1 l of an overnight culture grown in MGY (1.34% yeast nitrogen base, 1% glycerol and 4×10^−5^% biotin) media. Aeration was kept above 20% O_2_ saturation and the glycerol fed-batch was performed for 5 h feeding ∼500 ml of 50% (v/v) glycerol. Protein expression was induced by addition of 3.6 ml/h l (∼1000 ml) methanol for 48 h. Cells were harvested by centrifugation (5.000 xg, 10 min, 4°C), flash-frozen in liquid nitrogen and stored at −80°C until further use. Under these conditions approximately 1–1.4 kg of wet cell mass could be obtained.

### Expression of GFP fusion proteins in shaking flask cultures

Clones either expressing BSEP-GFP or MDR3-GFP were inoculated in 2 l shaking flasks containing 0.5 l of MGY media and shaken overnight at 30°C and 220 rpm. Protein expression was induced with methanol by harvesting the cells in sterile centrifuge buckets (5.000 xg, 10 min, 4°C) and re-suspended in 0.5 l methanol-containing media (MMY). 24 h after induction, methanol was added to a final concentration of 0.5% and after 48 h the cells were harvested (5.000 xg, 10 min, 4°C), flash-frozen in liquid nitrogen and stored at −80°C until further usage.

### Confocal fluorescence microscopy of GFP fusion proteins

*P. pastoris* cells expressing either BSEP-GFP or MDR3-GFP were directly spotted onto microscope slides coated with poly-L-lysine (Thermo Scientific) from shaking flasks and mounted with a coverslip. Images were acquired using an Olympus FV1000 confocal laser scanning microscope equipped with a 60×UPLSAPO objective (N.A. 1.35). GFP was excited at 488 nm and emission was recorded at 500 nm–600 nm.

### Preparation of crude membrane vesicles for protein purification

100 g batches of *P. pastoris* cells expressing BSEP or MDR3 were thawed on ice, washed with ddH_2_O and re-suspended at a concentration of 0.5 g cells/ml in homogenization buffer containing protease inhibitor cocktail (Roche). Cells were disrupted by two passages through a pre-cooled TS Series Cell Disrupter (Constant Systems) at 2.5 kbar. After cell debris was spun down by two centrifugation steps (15 min at 5,000 xg, 4°C and 30 min at 15,000 xg, 4°C), crude membrane vesicles were prepared by ultracentrifugation for 1 h at 125,000 xg, 4°C. Membrane vesicles were re-suspended in buffer A (50 mM Tris-HCl pH 8.0, 75 mM NaCl, 30% (v/v) glycerol) and flash frozen in liquid N_2_.

### Solubilization screen via the Dot Blot technique

Membranes were thawed on ice and solubilized in 200 µl buffer A. Membrane concentration was kept at 5 mg/ml during solubilization and detergents were used at a concentration of 1% (w/v) or higher according to their critical micellar concentration (cmc). A complete list of the used detergents is provided in Table S1 in File Supplementary Information. Samples were solubilized for 1 h at 4°C on a rotator, centrifuged (100.000 xg, 30 min, 4°C) and the supernatant was supplemented with SDS sample buffer. The samples were heated to 65°C for 10 min and 3 µl were spotted onto a dry nitrocellulose membrane. After extensive drying of the sample, the membrane was blocked for 1 h in TBS-T with 5% (w/v) milk powder and then probed with a 1∶2000 dilution of the respective primary antibody. Dot blots were quantified using the GeneTools software (Syngene).

### Fluorescence-detection size-exclusion chromatography (FSEC)

BSEP-GFP or MDR3-GFP containing membranes were solubilized in detergents based on the results of the dot blot analysis. 100 µl of the solubilized sample was applied to a Biosep SEC-S4000 size-exclusion chromatography column (Phenomenex) connected to a HPLC system (Hitachi) equipped with a fluorescence detector (L-2485, Hitachi), which was equilibrated in running buffer (50 mM Tris-HCl, pH 8.0, 150 mM NaCl, 15% (v/v) glycerol and 0.02% (w/v) β-DDM). The UV absorption of the proteins was followed at 280 nm and for online fluorescence detection, the GFP tag was excited at λ_ex_ = 470 nm to improve the signal to noise ratio and fluorescence emission was detected at λ_em_ = 512 nm.

### Solubilization and Purification of MDR3 and BSEP

The purification of MDR3 and BSEP was performed by tandem-affinity purification (TAP) consisting of an immobilized metal ion affinity chromatography (IMAC) step followed by a calmodulin binding peptide affinity purification (CBP). All procedures were carried out at 4°C. Crude membrane vesicles equivalent to 100 g wet cells were thawed at 4°C, diluted to a final concentration of 5 mg/ml total protein with buffer A as determined by the Coomassie Plus Assay (Pierce) and solubilized in 1% (w/v) of Fos-choline-16 or other detergents for 1 h at 4°C (for cmc values see Table S1 in File Supplementary Information). Non-solubilized membrane vesicles were removed by centrifugation at 100.000 xg, 4°C for 1 h. The supernatant supplemented with 20 mM imidazole was loaded onto a Ni^2+^-loaded HiTrap Chelating column (5 ml, GE Healthcare) and washed with 10 column volumes of buffer A supplemented with 20mM imidazole and typically 2.5×cmc of detergent. Proteins were eluted in one step with buffer B (50 mM Tris-HCl pH 8.0, 75 mM NaCl, 200 mM imidazole, 20% (v/v) glycerol) supplemented with 2.5×cmc detergent. The IMAC eluate was diluted 5-times with CaCl_2_ binding buffer (50 mM Tris-HCl pH 8.0, 150 mM NaCl, 1 mM MgCl_2_, 2 mM CaCl_2_ and 20% (v/v) glycerol) containing 2.5×cmc detergent, applied to 4 ml calmodulin affinity resin equilibrated in CaCl_2_ binding buffer and incubated with the calmodulin resin over night at 4°C on a rotator. The resin was transferred into a gravity flow column and washed with 10 column volumes of CaCl_2_ binding buffer containing 2.5×cmc detergent. The proteins were eluted with 3 bed volumes of EGTA elution buffer (2 mM EGTA, 50 mM Tris-HCl pH 7.4, 150 mM NaCl, and 20% (v/v) glycerol) supplemented with 2.5×cmc detergent. The purified protein was directly used for ATPase activity or further concentrated using an Amicon Ultra-15 filter (Millipore) with a cut-off of 100 kDa, aliquoted, snap frozen in liquid nitrogen and stored at −80°C. Aliquots of the sample were analyzed by Coomassie blue stained SDS-PAGE and immunoblotting.

### ATP Agarose binding assay of BSEP

To test the ability of detergent solubilized BSEP to bind ATP, 25 µl of a 1∶1 slurry of C8-linked ATP-agarose resin (Sigma) equilibrated in buffer A was added to 20 µg of purified BSEP in the detergent to be examined and incubated at 4°C on a rotator. After 1 h, the resin was pelleted by centrifugation (8200 xg, 2 min, 4°C) and the resin was washed three more times with 250 µl of buffer A supplemented with 2.5×cmc of the detergent. Bound proteins were eluted in SDS sample buffer by heating the resin to 65°C for 20 min. The pellet samples were subjected to SDS-PAGE and analyzed by immunoblotting.

### ATPase activity measurements of MDR3

The ATPase activity of MDR3 was examined with the malachite green assay by determination of released free inorganic orthophosphate as described previously [47]. Reactions were performed in a total volume of 100 µl in buffer C (50 mM Tris-HCl pH 7.4, 50 mM NaCl, 15% (v/v) glycerol) containing 2.5×cmc detergent and 10 mM MgCl_2_. 5 – 20 µg purified, detergent-soluble MDR3 was used. The reaction was started by typically adding 2 mM ATP at 37°C and stopped at appropriate time points by the addition of 25 µl of the reaction into 175 µl of 20 mM ice-cold H_2_SO_4_. Subsequently, 50 µl dye solution (0.096% (w/v) malachite green, 1.48% (w/v) ammonium molybdate, and 0.173% (w/v) Tween-20 in 2.36 M H_2_SO_4_) was added. After 15 min the amount of free phosphate was quantified spectroscopically by measuring the absorption at 595 nm. For subsequent data evaluation, all appropriate controls were performed and subtracted. For calibration of free phosphate concentrations a Na_2_HPO_4_ standard curve was used. For substrate stimulated ATPase activity, purified MDR3 was incubated with the equal volume of 2–5 mM lipid stock solution at room temperature for 20 min and sonified for 30 s to facilitate the incorporation of lipids into the detergent-protein micelles. The lipid-protein sample was stored on ice until further usage.

## Results

### Cloning and Expression of human BSEP and MDR3 in *P. pastoris*

**Figure 1:**
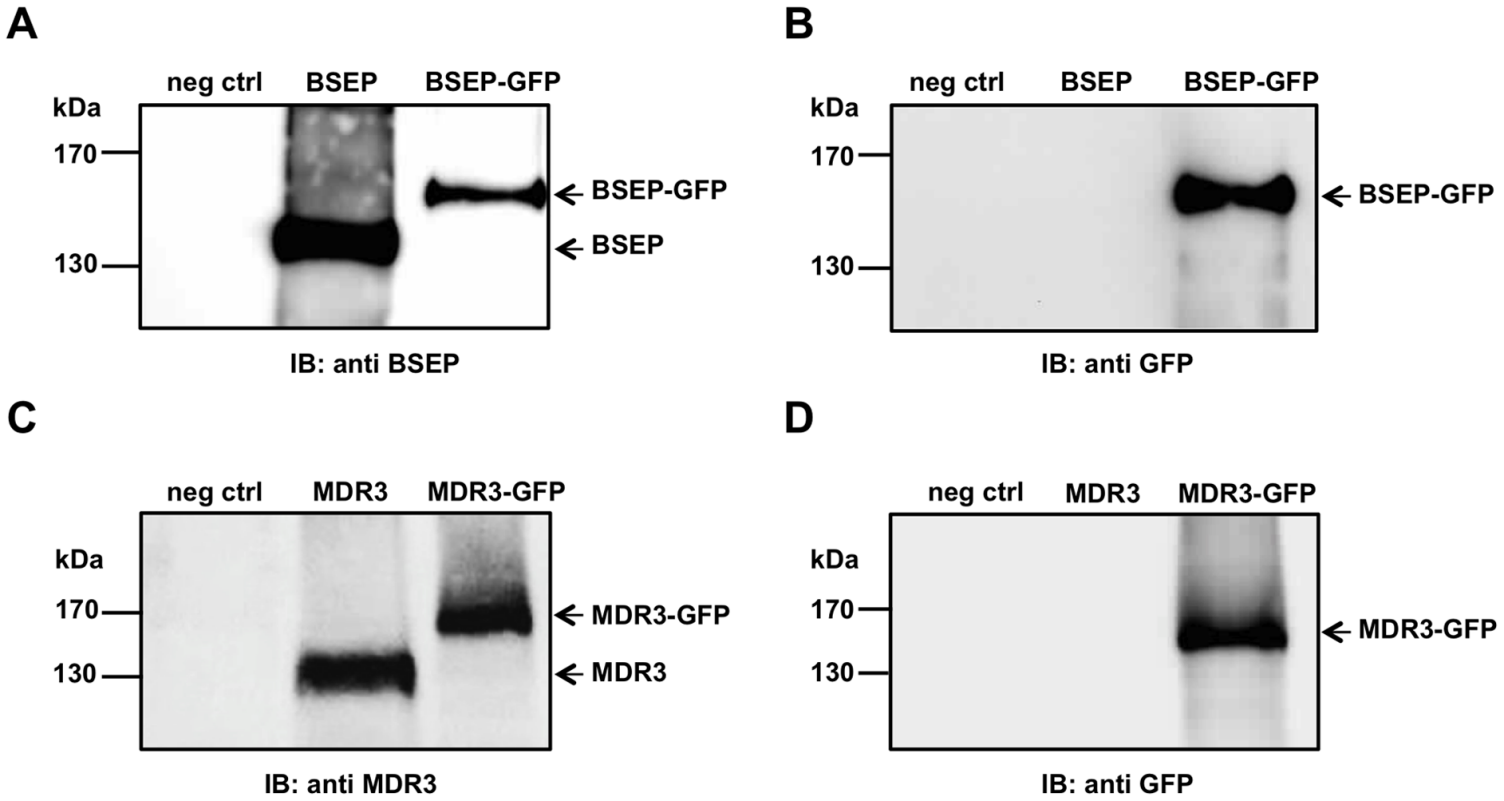
Human BSEP and MDR3 expression in *Pichia pastoris.* **A** 5 µg of membranes derived from *P. pastoris* cells carrying the empty expression plasmid pSGP18 (neg ctrl), BSEP or BSEP-GFP were subjected to SDS-PAGE and immunoblotting (lanes from left to right). The negative control (left lane) did not react with the monoclonal antibody (F-6), while BSEP (middle lane) and BSEP-GFP (right lane) could be detected by the same antibody. **B** Identical samples were probed with a monoclonal GFP antibody. The negative control (left lane) as well as BSEP (middle lane) showed no signal with anti-GFP antibody, while BSEP-GFP could be detected (right lane). **C** In case of MDR3 the negative control (left lane) showed no signal with the monoclonal antibody C219; MDR3 (middle lane) as well as MDR3-GFP (right lane) could be detected with the monoclonal antibody C219. **D** Identical MDR3 samples were probed with a monoclonal GFP antibody. The negative control (left lane) as well as MDR3 (middle lane) showed no signal with anti-GFP antibody, while MDR3-GFP could be detected (right lane). The position of the molecular weight markers are shown on the left.

For the expression of human BSEP and MDR3 in the methylotrophic yeast *P. pastoris* we used the expression plasmid pSGP18, which was used before to express 25 human ABC transporters in *P. pastoris* [34]. BSEP and MDR3 were not included in this study likely due to the inherent toxicity of the cDNAs, which hampers the cloning procedure and often results in the failure of obtaining suitable expression plasmids [13]. We custom modified the plasmid by introducing a 24 origin of replication for *S. cerevisiae* in its backbone and cloned the human *BSEP* and *MDR3* cDNA via homologous recombination into pSGP18-2µ. After transformation in *P. pastoris*, ten clones were tested for expression. A clone for each transporter was chosen for fermentation, which yielded about 1.0–1.4 kg of wet cell weight (wcw) in a typical fermentation. As can be seen by immunoblotting both wild-type proteins were expressed in *P. pastoris* (Fig. 1A and C, middle lanes). The wild-type proteins exhibited a distinct protein band at ∼130 kDa that cross-reacted with monoclonal antibodies against BSEP or MDR3. No signal was obtained using the empty plasmid as a control (see Fig. 1, neg ctrl).

### Localization and judging the quality of BSEP and MDR3 in *P. pastoris*

**Figure 2:**
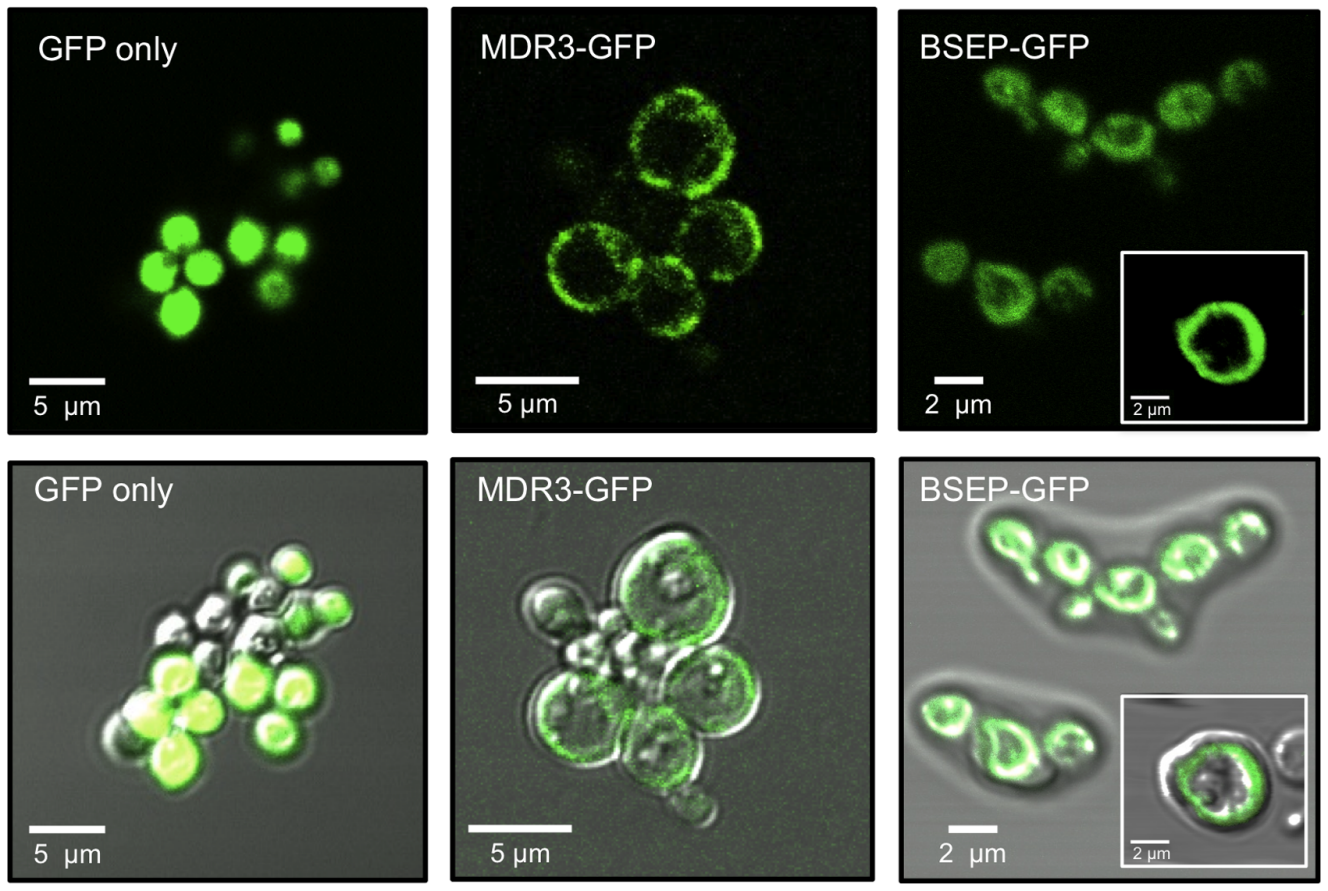
Fluorescence microscopy of BSEP-GFP and MDR3-GFP expressing *Pichia pastoris* cells. *P. pastoris* cells expressing GFP, BSEP-GFP or MDR3-GFP were harvested 48 h after induction and examined for GFP fluorescence (upper row) by confocal LSM. BSEP-GFP as well as MDR-GFP was located in the plasma membrane of *P. pastoris* cells in contrast to soluble GFP, which was homogenously distributed within the cell. Bottom row: merge of the GFP fluorescence and the Differential Interference Contrast (DIC) scans.

For the determination of the trafficking and localization of human BSEP and MDR3 in *P. pastoris* cells, we generated and expressed the corresponding GFP-fusion proteins, BSEP-GFP and MDR3-GFP. The C-terminal GFP-tag was confirmed by immunoblot analysis against GFP (Fig. 1B and D) as well as by a shift to a higher molecular weight visualized by antibodies against BSEP and MDR3, respectively (Fig. 1A and C, right lane). Both the fusion proteins migrated at ∼160 kDa. The correct trafficking of the GFP-fusion proteins to the plasma membrane of *P. pastoris* was checked by confocal laser scanning microscopy (Fig. 2, upper row). Induced cells expressing BSEP-GFP or MDR3-GFP showed clear ring-shaped fluorescence at the plasma membrane, which co-localized with the cell surrounding of the differential interference contrast (DIC) scan (Fig. 2, bottom row, merged pictures). As control only GFP was expressed in *P. pastoris* and the fluorescence was distributed homogenously within the cell, which leads to the conclusion that BSEP and MDR3 are processed and trafficked correctly in *P. pastoris*. Non-induced cells did not show any fluorescence (data not shown). We also employed sucrose density centrifugation of whole cell membranes containing BSEP or MDR3, which demonstrated co-localization of a plasma membrane marker with BSEP or MDR3, respectively (data not shown).

### Solubilization Screen via the Dot Blot technique

To find an appropriate detergent for membrane protein extraction, we tested over 100 different detergents for their ability to solubilize BSEP and MDR3 via dot blot analysis. These detergents covered all four classes: non-ionic (N), anionic (A), cationic (C) as well as zwitterionic (Z) (Table S1 in File Supplementary Information). Most of the detergents were used at a concentration of 1% (w/v). However, depending on the critical micellar concentration (cmc) other concentrations were also chosen when necessary (see Table S1 in File Supplementary Information).

**Figure 3:**
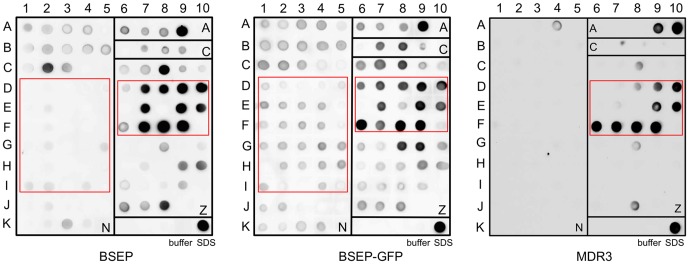
Solubilization screen of human BSEP and MDR3 using Dot Blot. Solubilization screen of *P. pastoris* membranes containing BSEP (**A**), BSEP-GFP (**B**) or MDR3 (**C**) with over 100 different detergents were analysed regarding the solubilization efficiacy. The solubilized protein was spotted onto a nitrocellulose membrane and examined via dot blotting with BSEP or MDR3 specific monoclonal antibodies (F-6 and C219). Compared areas are marked with a red box. All dot blots were performed in duplicate.

Membranes were solubilized for 1 h at 4°C, subsequently centrifuged and the supernatant was spotted on the dot blot membrane. For BSEP, we tested solubilization of the wild-type protein as well as the GFP-fusion protein, to investigate if the GFP-tag had any influence on the solubilization. Therefore, the BSEP-GFP fusion protein was fermented the same way as the wild-type BSEP protein for comparison. As seen in Fig. 3A and 3B, BSEP-GFP could be extracted more efficiently than BSEP by maltosides and glucosides (D-I 1-5). Furthermore, some differences can be seen in a more efficient extraction of BSEP-GFP in Fos-choline-unisat-11-10 and Fos-choline-8 (G8 and G9). Despite this, there are large similarities between BSEP and BSEP-GFP, in fact only the Fos-choline and Cyclofos detergents were able to solubilize both proteins in large quantities (Fig. 3A and B, D7-10, E7-10, F7-9). Also the Anapoe detergents (A-C 1-5) solubilized BSEP, but to a lesser extent. Furthermore, the anionic detergent dodecanoyl sarcosine (A9) as well as the zwitterionic detergents Anzergent® 3–14 (C8) and 2-carboxy-ω-heptadecenamidopropyldimethylamine (J8) resulted in strong signals in the dot blot.

In contrast to BSEP, we observed that only lipid-like detergents like the Fos-choline series (Fig. 3C, E9-E10, F6-F9, G8) and Cyclofos series (Fig. 3C, D8-D10) were able to solublize MDR3 in high amounts. In addition, the anionic detergents sodium dodecanoyl sarcosine (A9) and n-dodecyl-β-iminodipropionic acid (A10) were also able to solubilize MDR3. Furthermore, very low amounts of MDR3 were solubilized by Anzergent® 3-14 (C8) and 2-carboxy-ω-heptadecenamidopropyldimethylamine (J8). None of the Anapoes except Anapoe-58 (A4), none of the glucosides, none of the thio-maltosides, none of the maltosides or any other series of detergents showed a signal indicating that MDR3 was completely resistant to solubilization. In the case of MDR3, we did not analyze the GFP-fusion protein, because wild-type MDR3 displayed a substrate-induced ATPase activity (see below). Thus, the dot blot based solubilization screen revealed that only the lipid-like and more “harsh” detergents of the Fos-choline and Cyclofos series were able to solubilize both, BSEP and MDR3, in a near quantitative manner. For a quantification of the dot blots see Figure S1 in File S1.

### Fluorescence-detection Size Exclusion Chromatography of selected detergents

Based on this analysis, the result of selected detergents used for the solubilization of BSEP and MDR3 were examined by fluorescence-detection size exclusion chromatography (FSEC). SEC is a common tool for monitoring the monodispersity and stability of proteins. In combination with a fluorescence detector, we were able to ascertain a high number of detergents using the GFP fusion proteins as reporter. This strategy requires only nanogram quantities of non-purified GFP-fusion protein by directly using solubilized membrane proteins in the detergent to be investigated. Our criteria for FSEC profiles in terms of monodispersity and stability were a sharp and symmetrical peak, no or only a small peak in the void volume or no signal corresponding to free GFP, which would indicate degradations of the fusion protein (for a FSEC profile of free GFP see Figure S2 in File S1).

**Figure 4:**
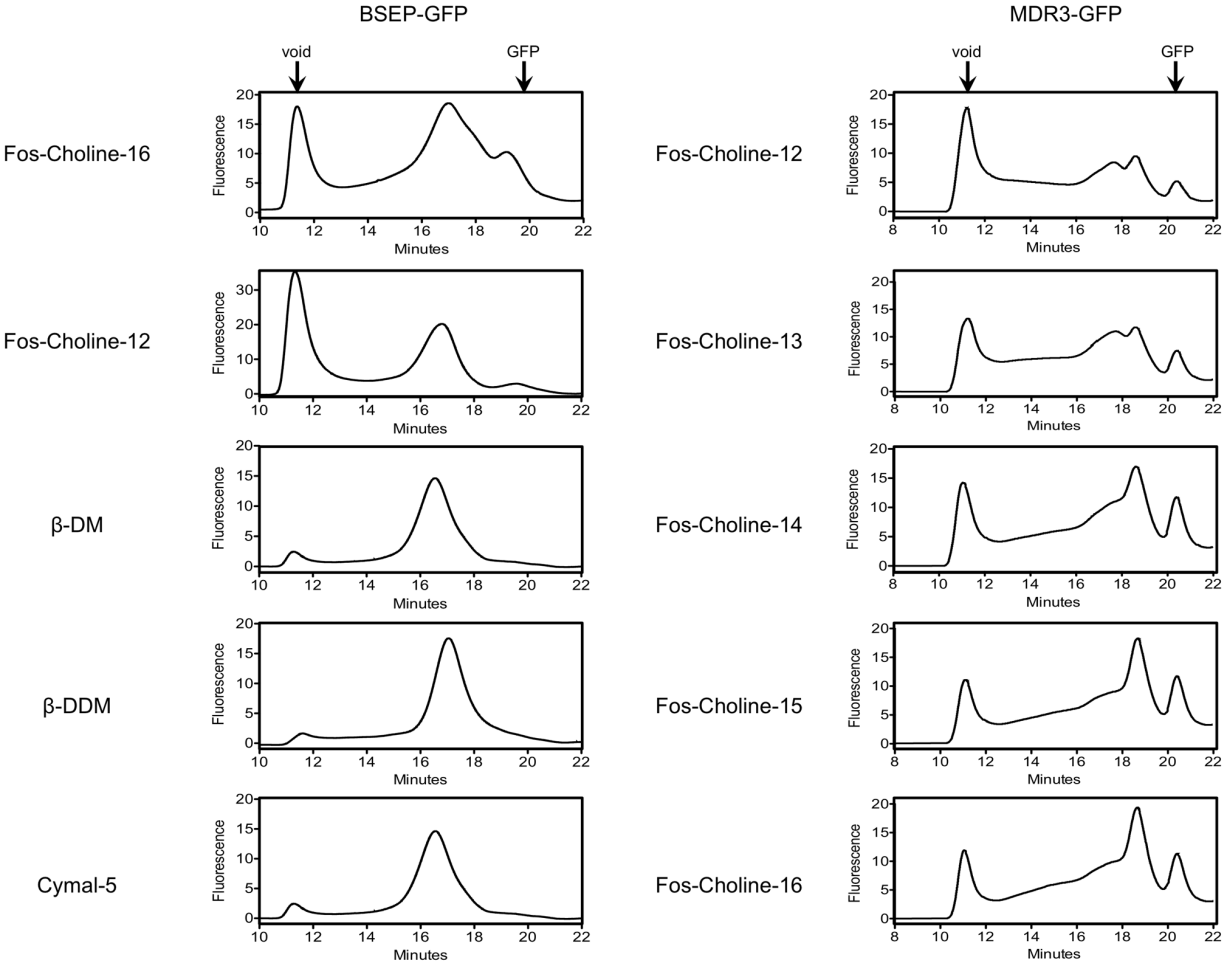
Detergent screening utilizing FSEC. FSEC analysis of BSEP-GFP (**A**) in five representative detergents and MDR3-GFP in five detergents (**B**). The arrows indicate the estimated elution position of the void volume and free GFP. Additional FSEC profiles are summarized in the supplementary material.

The Fos-choline series as well as some maltosides and other detergents (see Figure S3 in File S1) solubilized BSEP-GFP, although the latter only resulted a weak signal in the dot blot. Fos-choline 8 and 9 did not give a significant signal in FSEC. A reliable signal was only obtained in the case of Fos-cholines containing long acyl chains. The signal increased with increasing acyl chain length from 10 to 16 carbon atoms (Fig. 4A). BSEP-GFP eluted to a certain portion in the void volume in Fos-choline detergents (10–11 min retention time), especially in Fos-choline-12 (Fig. 4A) indicating aggregated protein. The main BSEP-GFP peak (between 16 and 17 minutes) became more non-symmetrical and more BSEP-GFP degradation product (∼20–21 min retention time, free GFP) was detected for detergents with longer acyl chains (Fig. 4A and Fig S3 in File S1). On the other hand, the maltosides gave sharp and symmetrical FSEC chromatograms and only very little aggregation was detected. This was very pronounced for β-DM, β-DDM and Cymal5. This implies that the protein was monodisperse and stable. Other detergents tested such as the anionic detergent sodium dodecanoyl sarcosine resulted in a non-symmetrical peak (Figure S3 in File S1). These observations, suggested that the length of the acyl chain of either group of detergents had a profound influence on the monodispersity and that an acyl chain length between 10 to 13 carbon atoms preserved the monodispersity of the transporters.

The FSEC profiles obtained for MDR3-GFP using the aforementioned detergents are summarized in Figure 4B and Figure S2 in File S1. None of the detergents showed perfect monodisperse peaks. The anionic detergent n-dodecyl-β-iminodipropionic acid was able to solubilize MDR3 (Figure S2 in File S1), however, the FSEC peak resulted in a major signal in the void volume of the SEC (molecular weight>1 MDa). This suggested aggregation. Sodium dodecanoyl sarcosine and the group of Cyclofos detergents showed a very inhomogeneous SEC profile (Figure S2 in File S1). The lipid-like Fos-cholines solubilized MDR3 with high efficiency nearly to the same extent as the SDS sample, which was used as control for solubilization efficiency (Fig. 4B). Importantly, the length of the acyl chain had again an impact on the monodispersity and stability of MDR3-GFP. The longer the acyl chain became, the more symmetrically the MDR3-GFP peak was observed (FC-16>FC-15>FC-14>FC-13>FC-12). The most promising result of solubilization efficiency and monodispersity was obtained for FC-16, so that all further experiments such as purification and ATPase acitivity were performed in this particular detergent.

### Purification of the human ABC transporter MDR3 and BSEP

For functional analysis, we purified both transporter in the detergents, which showed the most promising results in the dot blot and FSEC analysis. The procedure we applied for the purification of human BSEP and MDR3 was established by Wang *et al*. based on the purification of the human ABC-transporters ABCG5/G8 and ABCC3 expressed in *P. pastoris* [34], [46] and is described in detail in “Materials and Methods”. MDR3 and BSEP both contain a tandem affinity tag consisting of a calmodulin binding-peptide tag (CBP-tag) and a 6xhis-tag at their C-termini. Briefly, BSEP and MDR3 were purified by immobilized metal-ion affinity chromatography (IMAC) and calmodulin affinity resin (CBP) after solubilization of crude membranes in the appropriate detergent isolated from fermenter cultures.

**Figure 5:**
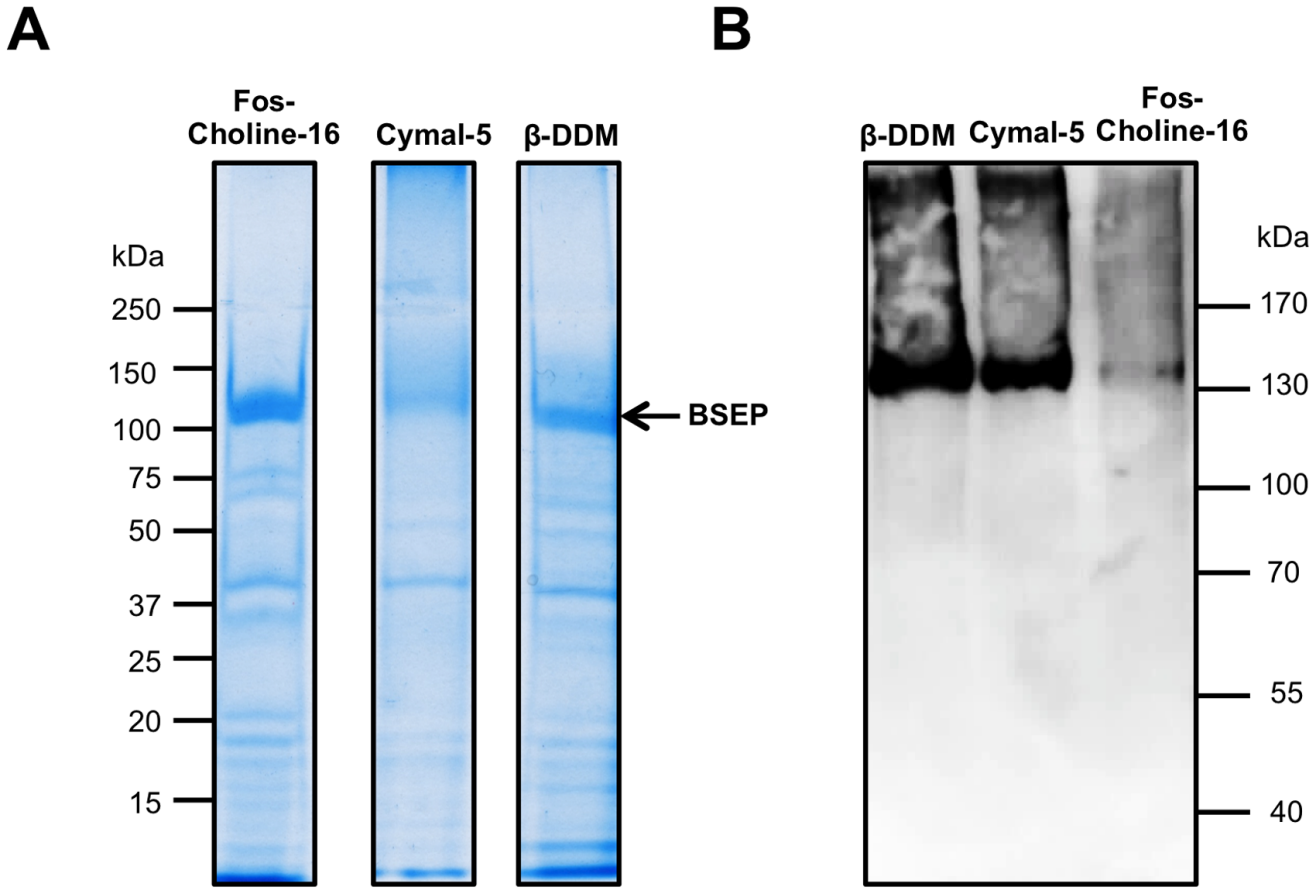
Purification and nucleotide binding of human BSEP. **A** Coomassie Brilliant Blue-stained SDS-PAGE of purified BSEP solubilized in Fos-choline-16 or in β-DDM and Cymal5, which were exchanged after solubilization. Molecular weight markers are indicated on the left. **B** Purified BSEP in all three detergents was incubated with ATP-agarose and bound protein was eluted in SDS sample buffer and examined with immunoblotting with a monoclonal antibody (F-6). BSEP signals could be detected in β-DDM and Cymal5, but not in Fos-Choline-16, indicating only binding to ATP in maltosides.

We chose Fos-choline-16 as detergent of choice for solubilization of BSEP, because of its high efficacy. During the purification process, we exchanged the detergent on the CBP affinity column to maltoside detergents (e.g. β-DDM and Cymal-5), which according to the FSEC profiles corresponded to monodisperse protein (Fig. 4A). BSEP could be purified and yielded ∼1 mg of protein from solubilized membranes of 100 g (wcw) of *Pichia* cells with a purity of roughly 75% (Fig. 5A).

**Figure 6:**
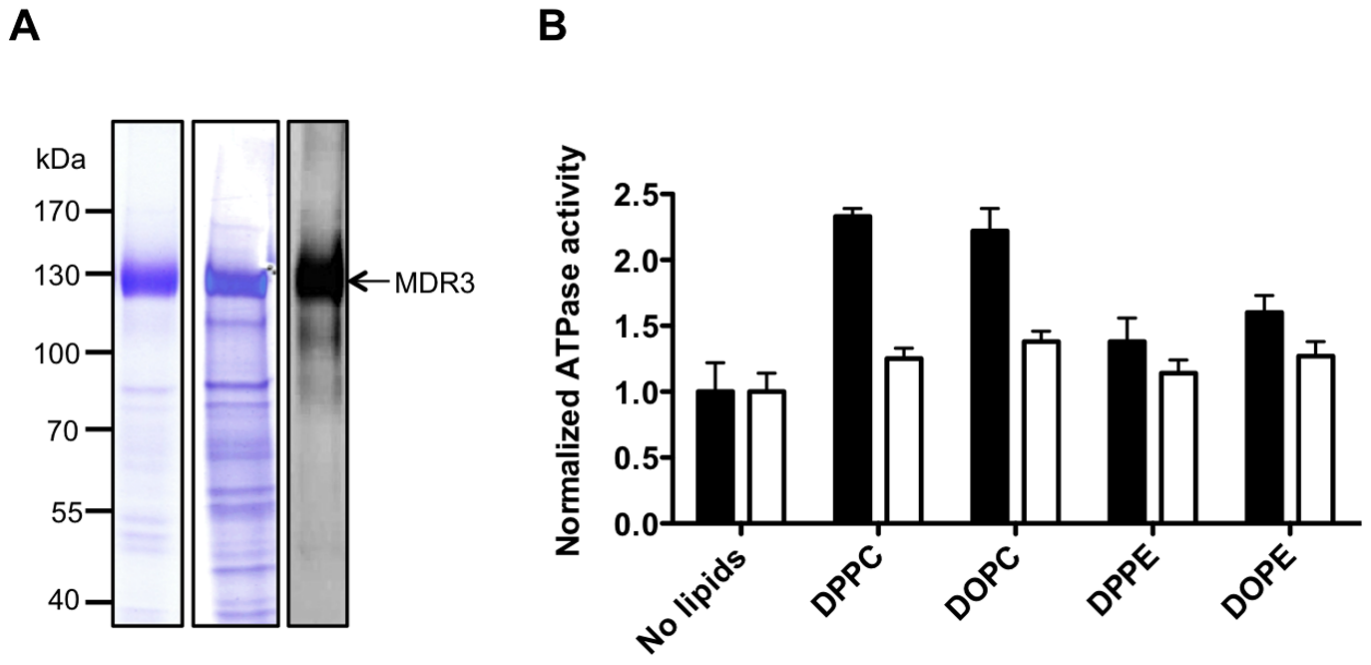
Characterization of purified human MDR3 in Fos-choline-16. **A** Coomassie Brilliant Blue-stained SDS-PAGE and immunoblot using an anti-MDR3 antibody of purified MDR3 wild-type and the MDR3 EQ/EQ-mutant via TAP. Molecular weight markers are shown on the left. **B** Normalized ATPase activity of MDR3 wild-type (black) and of an ATPase deficient mutant (E558Q E1207Q, white) in FC-16 without and with different phospholipids. The ATPase activity of three independent MDR3 purifications was determined ± SD (n = 3).

MDR3 was solubilized with Fos-choline-16 and purified via an identical tandem affinity approach. The MDR3 transporter was visualized on a Coomassie blue-stained SDS-gel and further identified by immunoblot analysis (Fig. 6A). We obtained ∼6 mg of highly purified protein from 100 g yeast cells with a purity of more than 90% as judged by SDS-PAGE analysis.

### Binding of solubilized human BSEP to ATP-Agarose

BSEP was tested for ATPase activity in detergent solubilized state, but no reliable activity could be detected. Therefore, we investigated the capability of BSEP to bind to ATP coupled to agarose beads (ATP-beads) in the detergent-solubilized state, which would indicate that the protein is in a state where the nucleotide can bind, but the conformation is likely locked in a non-productive state, which inhibits hydrolysis. As shown in Figure 5B BSEP purified in Fos-choline-16 was not eluted from the ATP-beads after incubation suggesting that BSEP cannot bind to ATP in Fos-choline. Maltosids are known as mild detergents and often find usage to preserve the functionality of the membrane protein such as LmrA [47]. Accordingly, we solubilized BSEP with Fos-choline-16 and exchanged the detergent to β-DDM or Cymal-5 during purification. In these two detergents, BSEP bound to the ATP-beads. This result is in agreement with the FSEC results in those detergents (Fig. 4A).

### ATPase Activity of purified human MDR3

We further examined whether purified MDR3 exhibits ATPase activity that could be stimulated by its natural substrate phosphatidylcholine (PC) lipids. For this purpose we added two synthetic PC lipids (DPPC and DOPC) to the purified protein and measured the ATPase activity at 37°C up to 60 min (Fig. 6B). Under these conditions, we observed an approximately 2.5 fold stimulation of ATPase activity. Because co-purification of contaminating ATPases cannot be excluded, we cloned an ATP hydrolysis deficient mutant by introducing two point mutations and purified the mutant as described for the wild-type protein. The exchange of Glu to Gln in the highly conserved Walker B motif (ΦΦΦΦDE, where Φ can be every hydrophobic amino acid) of MDR3 prevents hydrolysis of ATP. The ATPase inactive mutant (E558Q, E1207Q, further called EQ/EQ mutant) exhibited basal ATPase activity comparable to the wild-type protein. This suggested that the observed activity was derived from co-purified ATPases. However and most important, no stimulation of activity was observed in the presence of PC lipids. ATPase activity of ABC transporters is often stimulated after addition of lipids. To demonstrate that the increased ATPase activity of MDR3 is caused by a substrate-specific and not by a conformational stabilization effect of PC lipids, we added DPPE and DOPE lipids to MDR3 wild-type as well as to the ATPase-deficient EQ/EQ mutant. The MDR3 wild-type ATPase activity is slightly increased by a factor of 1.4 for DPPE and 1.6 for DOPE, whereas the ATPase activity of the EQ/EQ mutant are not increased compared to PC added ATPase activity. The data demonstrates a substrate-specific ATPase activity of 15 nmol/min per mg MDR3 wild-type in comparison to the DOPE-stimulated ATPase activity. We ascertained that the stimulation of ATPase activity is MDR3 specific by PC and indicated that MDR3 is functional in the detergent-solubilized state with respect to its capability to bind and hydrolyze ATP.

## Discussion

In this study, we presented a high-throughput detergent screening and purification approach for the human liver-localized ABC transporters BSEP and MDR3 expressed in the methylotrophic yeast *Pichia pastoris*. This expression host has all the advantages of other eukaryotic expression systems, such as post-translational modifications or trafficking machinery. However, the overexpression per cell is only moderate and therefore requires fermentation to compensate this by high biomass. This system was used before for expression trials of human ABC transporters, which showed its general applicability for this class of transporter. Especially ABC transporters of the liver like MDR1 (P-gp, ABCB1), ABCG2, ABCG5/G8 or ABCC1, ABCC3 and ABCC6 (MRP 1, 3 and 6) could be expressed and partially purified [34], [46], [48], [49], [50], [51]. Since BSEP and MDR3 were not included in this expression screen, we cloned these genes into the expression vector pSGP18. The cDNA of BSEP and MDR3 is unstable and cannot be cloned by conventional cloning in *E. coli* [45]. Therefore, we modified the pSGP18 vector. Both transporters as well as the GFP-fusion proteins were expressed without detectable degradation products (Fig. 1). To analyze whether processing and especially targeting of BSEP and MDR3 to the plasma membrane in *P. pastoris* occurs, we employed fluorescence microscopy. Fluorescence microscopy of heterologous expressed proteins, particularly with distinct destinations in the cell is a valuable tool to directly judge the quality of the overexpressed protein. These experiments revealed that both transporters were targeted correctly and no intracellular retention occurred. This adverts correct folding of BSEP and MDR3.

To date high-throughput methods are available to systematically screen a huge number of detergents in an appropriate time frame for their capability to solubilize the membrane protein of interest. We used a dot blot based solubilization screen on an analytical scale similar to approaches used for GPCRs heterologously expressed in *P. pastoris* or *E. coli* [36], [52]. We analyzed more than 100 different detergents covering all four classes of detergents. Only detergents of the Fos-choline as well as Cyclofos series were able to solubilize BSEP and MDR3 in a nearly quantitative manner. None of the maltosides, thio-maltosides or glycosides were able to solubilize BSEP and MDR3. Surprisingly, BSEP-GFP could be solubilized to some degree by those detergents suggesting that the GFP tag enhances solubilization. The zwitterionic Fos-choline and Cyclofos series are lipid-like detergents and possess a head group consisting of phosphocholine, but differ in the hydrophobic part as Fos-cholines have a plain acyl chain with varying number of carbon atoms and Cyclofos detergents additionally contain a cyclohexane ring at the omega position of the acyl chain. This result is in contrast to other used detergents for liver ABC transporters expressed heterologously in *P. pastoris*. ABCC3 was solubilized in β-DDM like ABCG5/G8 [34], [53]. MDR1 was solubilized in various detergents from *P. pastoris* membranes including β-DM, β-DDM, Lyso-PC, deoxycholic acid or Triton-X100 [48], [54], [55], [56], [57]. Despite the high degree of sequence identity between MDR1 and MDR3 (>85% homology to human MDR1, 80% to mouse MDR1), MDR3 behaves different, since it could not be solubilized with Triton-X100 (data not shown), which was used to crystallize mouse MDR1 [54]. Also ABCG2 was solubilized in β-DDM, but could only be solubilized in Fos-Choline-16 when expressed in *High Five* cells [46], [49].

GFP fusion proteins cannot only be used as quality marker for heterologous expression, but also as a tool to screen the influence of a detergent to the membrane protein using FSEC. We employed this technique to investigate those detergents more in detail that were successfully identified in the dot blot screen. The Fos-Choline series displayed a clear dependence on the acyl chain length, i. e. increasing the acyl chain length increased the monodispersity of the protein sample. For BSEP-GFP, we also tested some maltoside detergents, although the solubilization efficacy was moderate for BSEP-GFP as judged from the dot blot. All tested maltosides showed very monodisperse FSEC profiles with less aggregation and a symmetrical peak, e.g. with Cymal-5 or β-DDM. β-DDM in general is believed to be a mild detergent and is often used for solubilization, purification and crystallization trails. On the other hand, the Fos-cholines showed a large aggregation peak and with increasing acyl chain length, a BSEP-GFP degradation product was more visible indicating instability of the membrane protein, and the peak became more unsymmetrically. Nonetheless, we decided to use Fos-choline-16 for BSEP and MDR3 for solubilization because of its efficacy and its use for other ABC transporter like LmrA [47], BmrC/D [58] and the aforementioned ABCG2 [46].

We were able to purify MDR3 and BSEP for the first time yielding ∼6 mg and ∼1 mg of protein per 100 g of cells, respectively. This is in good agreement with ABCB1 (∼6 mg) or ABCC3 (∼9 mg) [34], [48]. BSEP is expressed at lower levels than MDR3 in *P. pastoris* and thus the yield is lower underlining the variance of expression of different proteins. Both transporters were purified by TAP from crude membranes, which resulted in a homogeneous preparation for MDR3 as judged by SDS-PAGE. In case of BSEP, the purity was not as high.

BSEP and MDR3 belong to the ABC transporter family and ATP hydrolysis drives translocation of bile salts or phosphatidylcholine. However, we could not detect any ATPase activity for BSEP in the detergent-solubilized state, neither basal nor substrate induced. To see whether BSEP was purified in a state, which at least allows binding of ATP, we employed ATP beads. Here, we could confirm that purified BSEP is able to bind ATP in the presence of β-DDM and Cymal-5. This indicates that at least the NBDs of BSEP are properly folded, which is a prerequisite for proper functioning and that Fos-choline-16 likely locks the protein in a binding-incompatible state, whereas β-DDM and Cymal-5 invert this state. For BSEP it is known, that its transport activity is depending on cholesterol [59]. Enrichment of *Sf*9 cell membranes expressing BSEP with cholesterol drastically increases its transport activity [60]. If cholesterol is bound to the transporter itself or is just required as a membrane component has not been clarified yet. In the yeast expression host however, ergosterol is the predominant sterol instead of cholesterol like in other mammalian cells. Both sterols differ by two additional double bonds (in the ring and in the tail) in the case of ergosterol. The striking dependence of transport activity of BSEP on cholesterol and the absence of this steroid in yeast might explain the lack of ATPase activity. However, even in the absence of cholesterol, BSEP is able to bind to ATP in the detergent-solubilized state.

MDR3 displayed a substantial ATPase activity. This observation was sustained by analysis of an ATP hydrolysis deficient EQ double mutant (E558Q, E1207Q). Generally, a mutation of the glutamine of the Walker B motif renders ABC transporters ATPase inactive. Here, we generated the double mutant to ensure that the observed stimulation of ATPases was not due to contaminating ATPases. The substrate specificity for MDR3 was already investigated using *S. cerevisiae* secretory vesicles or cell-culture based methods [16], [32], [61]. All experiments showed, that MDR3 translocates short chain PC lipids (C_8_) or long chain derivatives (C_16_), but not PE, sphingomyelin or ceramides.

Here, we demonstrate for the first time that the ATPase activity of detergent-solubilized MDR3 in the presence of phosphatidylcholine lipids could be stimulated by a factor of almost 2.5, while the EQ/EQ mutant did not display any stimulation. Furthermore, it was shown that the specificity resulted from the phosphatidylcholine headgroup. We proved that MDR3 ATPase activity is specifically stimulated by PC lipids and not by PE lipids, which differ only in the headgroup.

In summary, we demonstrate for the first time the expression of two human ABC transporters, MDR3 and BSEP, in the yeast *P. pastoris* and their correct targeting to the plasma membrane. BSEP could bind to ATP in detergent, but no hydrolytic activity could be detected. Furthermore, we established a purification procedure for human MDR3, which resulted in purified and functional protein. This study provides the foundation for further investigations of the human liver ABC transporters BSEP and MDR3.

## Supporting Information

File S1 **Combined file of supporting figures and tables.** Figure S1: Dot Blot quantification of BSEP (A), BSEP-GFP (B) and MDR3 (C). Average values from two independent dot blots are shown (n = 2) ± SD. Large errors for e.g. the Fos-Choline series resulted from saturation of the detector. The intensity of SDS was set to 100% and all other values were normalized to SDS. Black bars represent zwitter-ionic detergents, grey bars ionic detergents and white bars non-ionic detergents. Figure S2: FSEC profiles of free GFP and MDR3-GFP in selected detergents. The x-axis shows time in minutes, the y-axis fluorescence in arbitrary units. Figure S3: FSEC profiles of BSEP-GFP in selected detergents. The x-axis shows time in minutes, the y-axis fluorescence in arbitrary units. Table S1: Used detergents for solubilization of BSEP and MDR3 and Dot Blot analysis; N: Non-ionic detergents; Z: Zwitterionic detergents; A: Anionic detergents; C: Cationic detergents.
(DOCX)
