# Supplementary material for: Detergent Screening and Purification of the Human Liver ABC Transporters BSEP (ABCB11) and MDR3 (ABCB4) Expressed in the Yeast Pichia pastoris
Source: PLoS One. 2013 Apr 4;8(4):e60620. doi: 10.1371/journal.pone.0060620 (PMC3617136; doi:10.1371/journal.pone.0060620)
Supplement: File S1 — Combined file of supporting figures and tables. Figure S1: Dot Blot quantification of BSEP (A), BSEP-GFP (B) and MDR3 (C). Average values from two independent dot blots are shown (n = 2) ± SD. Large errors for e.g. the Fos-Choline series resulted from saturation of the detector. The intensity of SDS was set to 100% and all other values were normalized to SDS. Black bars represent zwitter-ionic detergents, grey bars ionic detergents and white bars non-ionic detergents. Figure S2: FSEC profiles of free GFP and MDR3-GFP in selected detergents. The x-axis shows time in minutes, the y-axis fluorescence in arbitrary units. Figure S3: FSEC profiles of BSEP-GFP in selected detergents. The x-axis shows time in minutes, the y-axis fluorescence in arbitrary units. Table S1: Used detergents for solubilization of BSEP and MDR3 and Dot Blot analysis; N: Non-ionic detergents; Z: Zwitterionic detergents; A: Anionic detergents; C: Cationic detergents. (DOCX) [file pone.0060620.s001.docx]

**Supplementary Information**

**Detergent Screening and Purification of the Human Liver ABC Transporters BSEP (ABCB11) and MDR3 (ABCB4) expressed in the yeast *Pichia pastoris***

Philipp Ellinger, Marianne Kluth, Jan Stindt, Sander H. Smits and Lutz Schmitt

Institute of Biochemistry, Heinrich Heine University, Düsseldorf. Germany

**Supplementary Information comprises**:

Supplementary Figures 1-3

Supplementary Table 1

**Supplementary Figure S1**

**
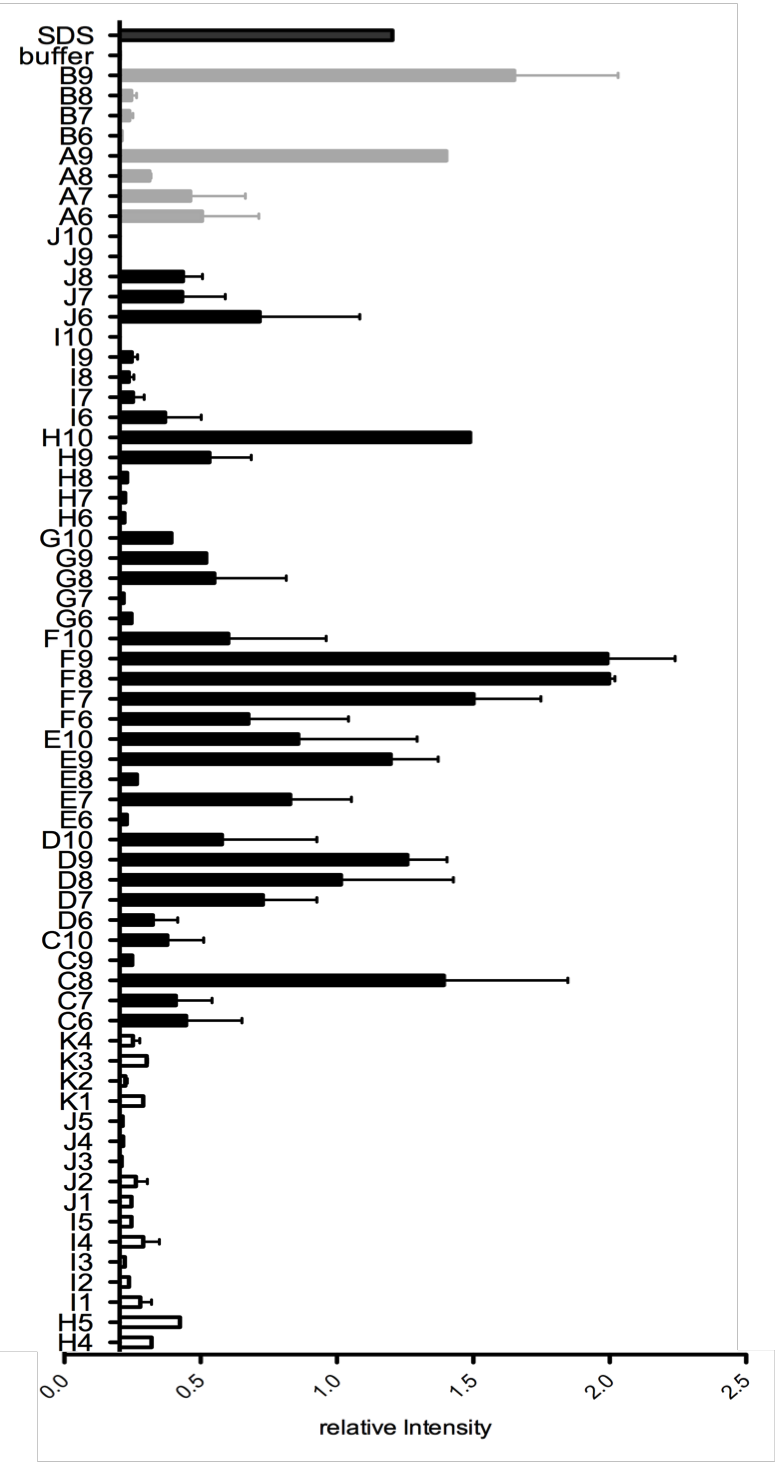
**

**A**

**
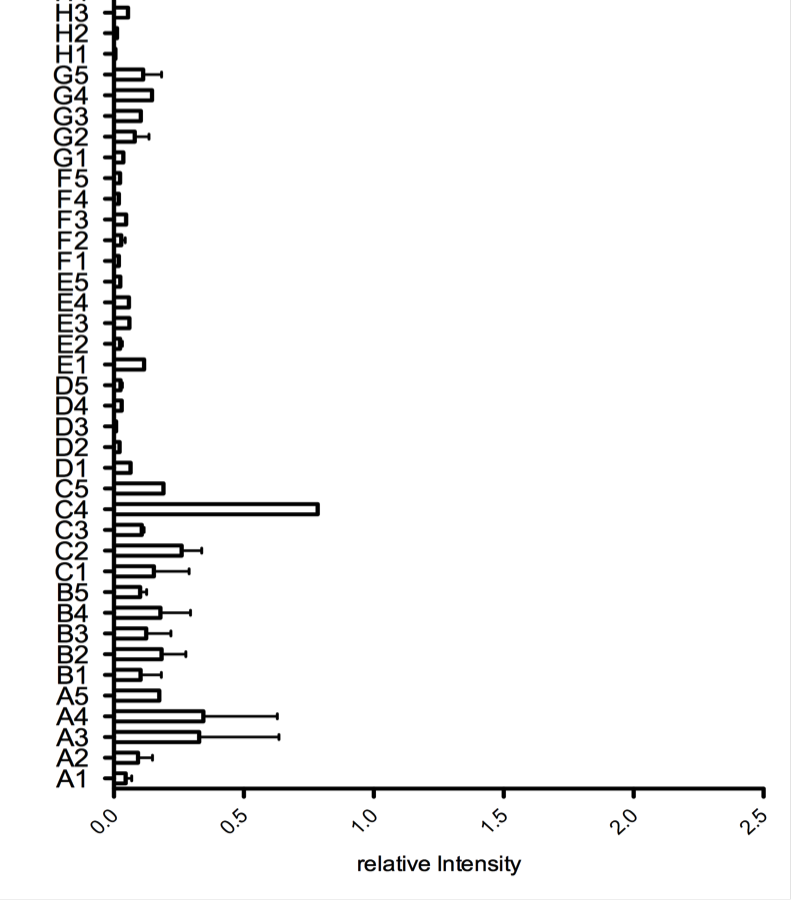
**

**
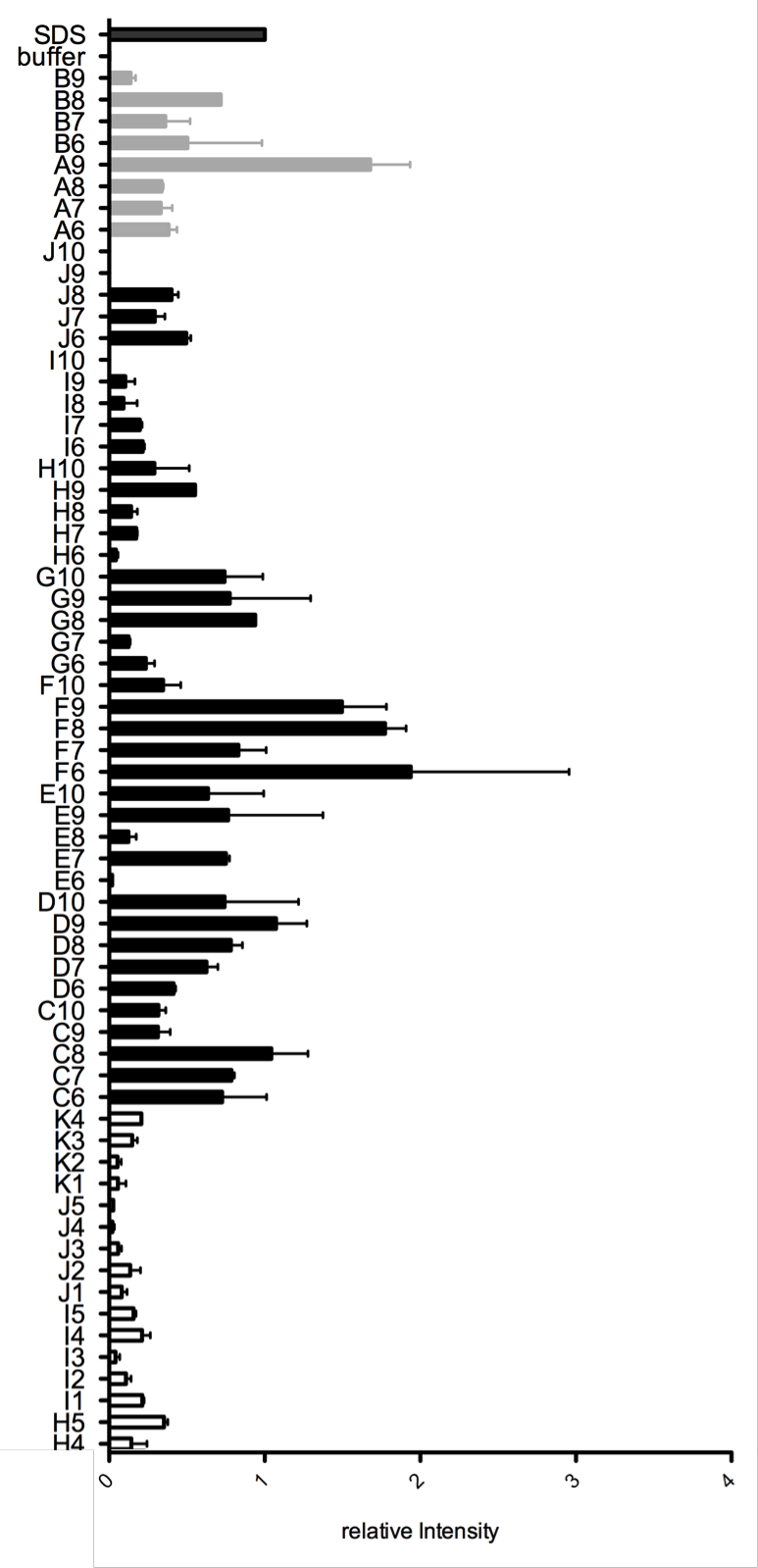
**

**B**

**
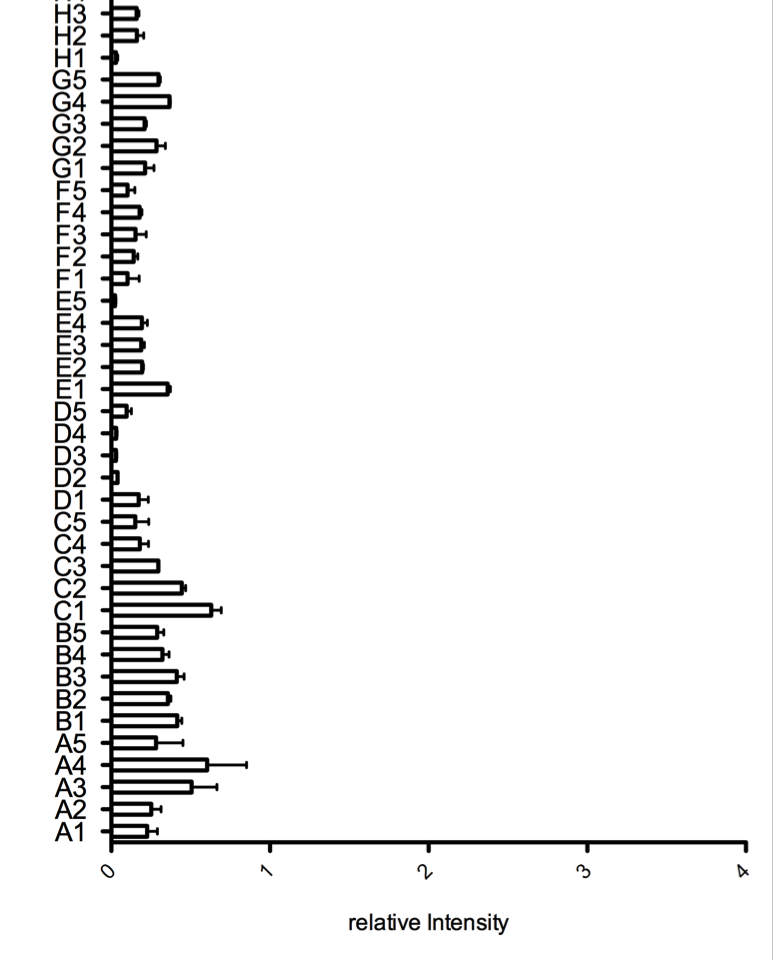
**

**
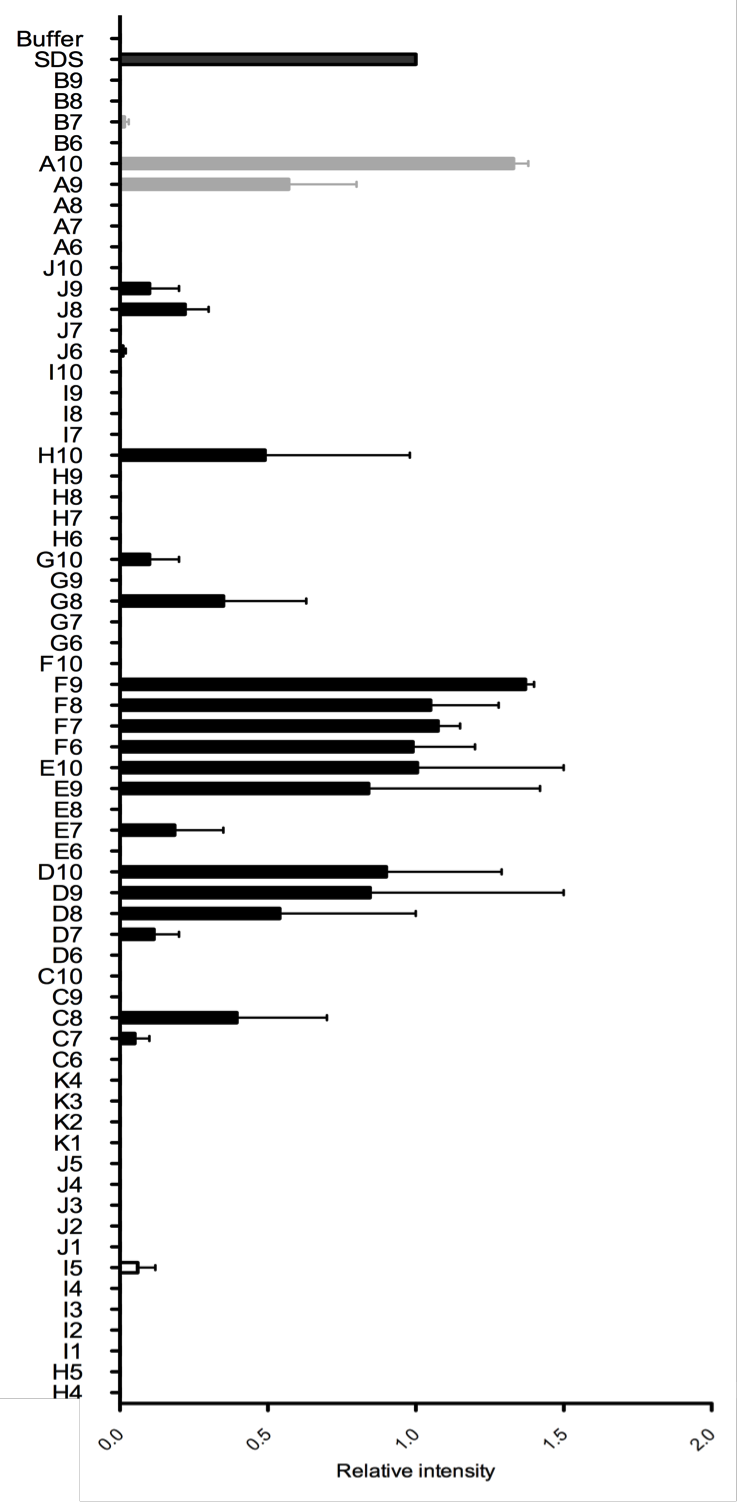
**

**C**

**
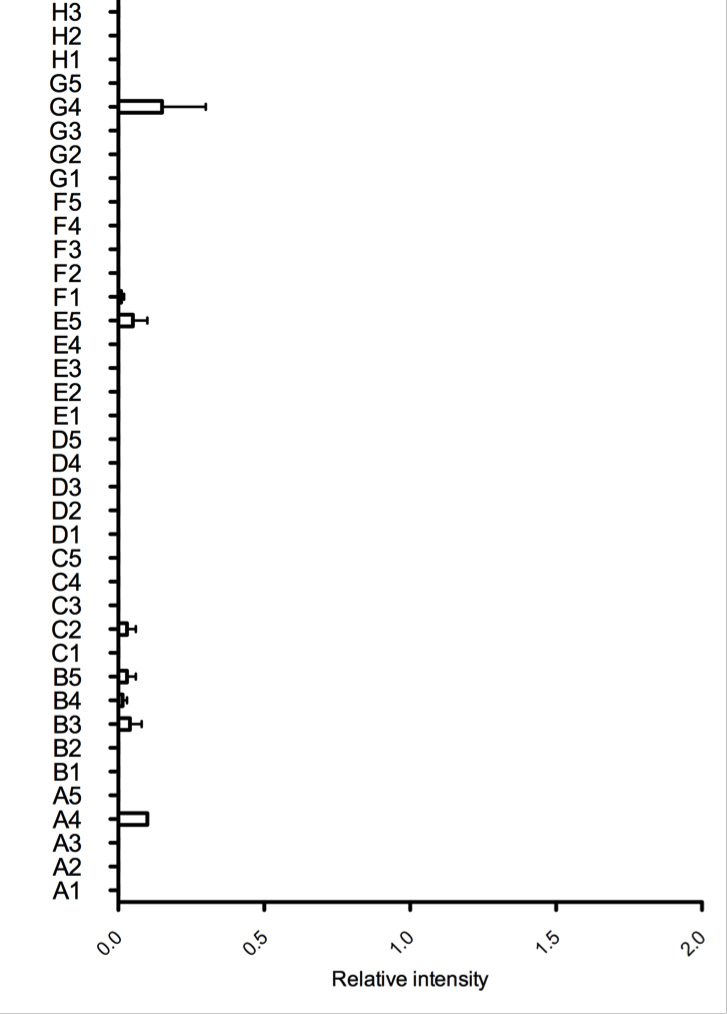
**

**Supplementary Figure S2**


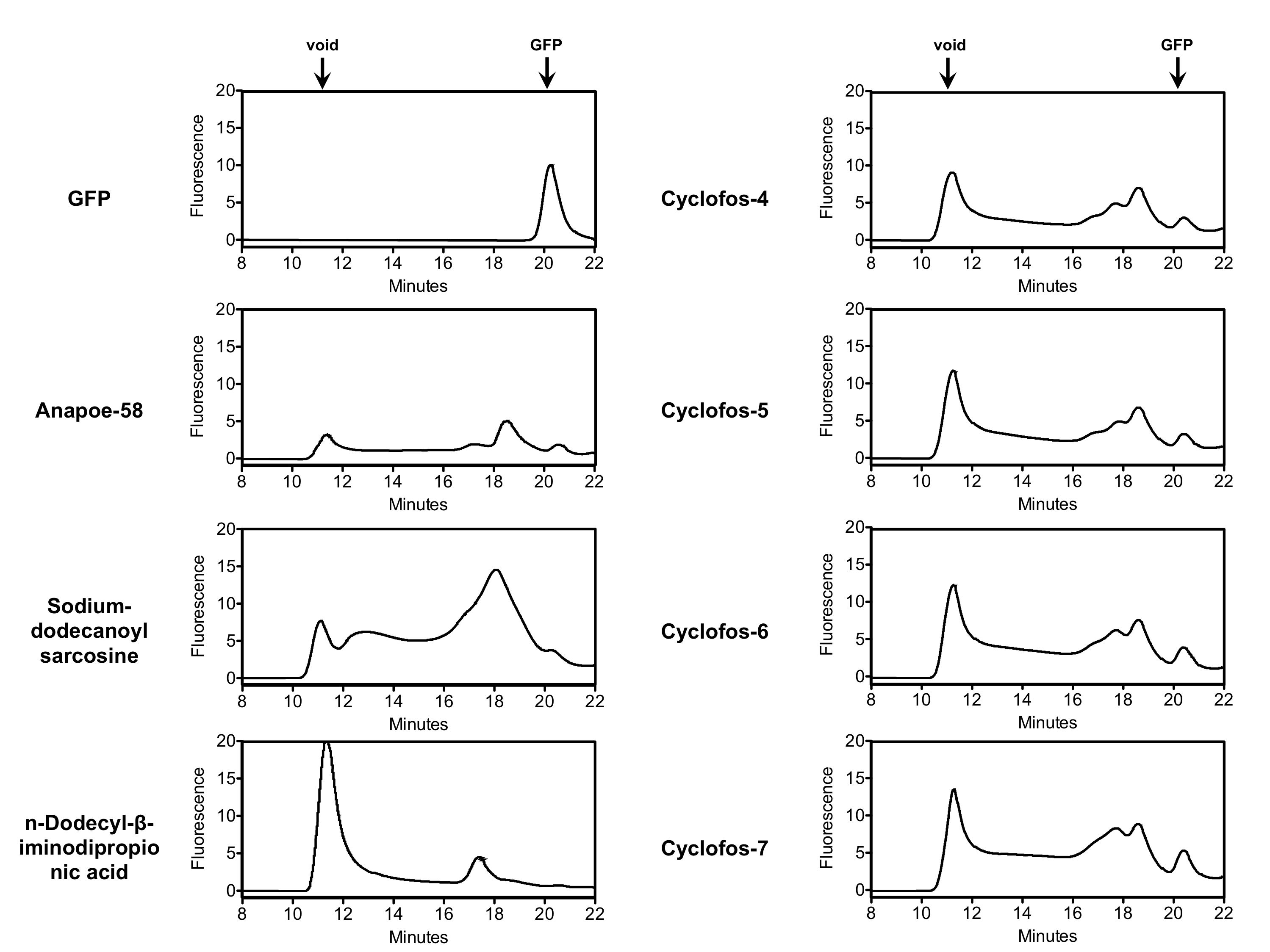


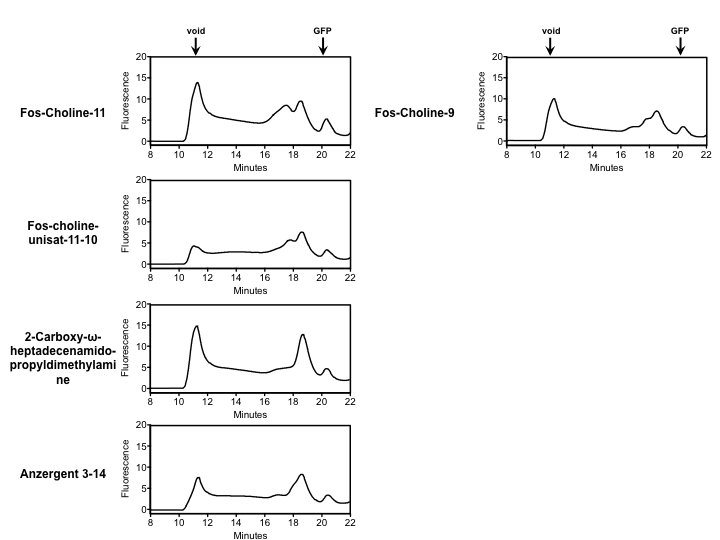


**Supplementary Figure S3**


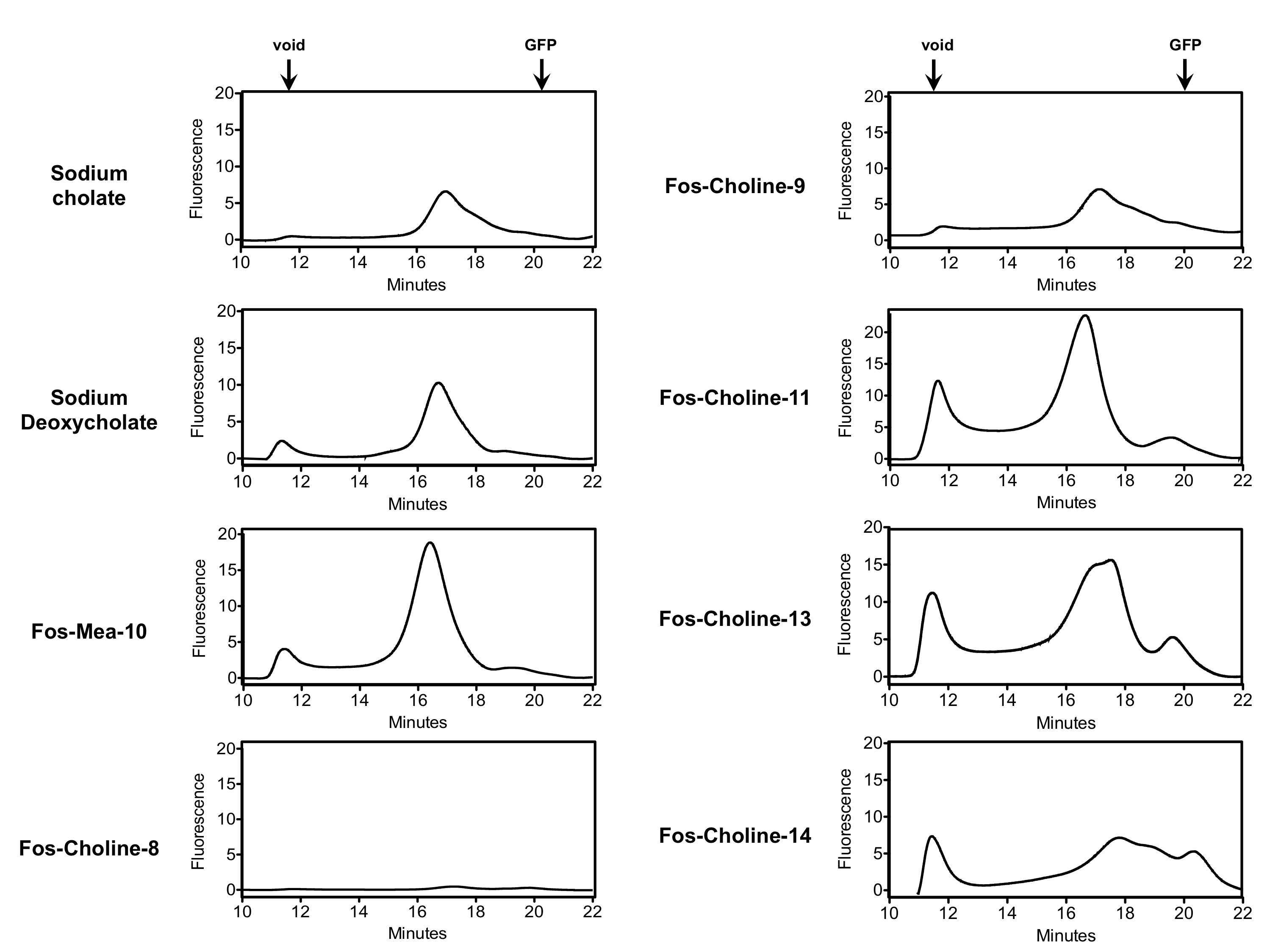


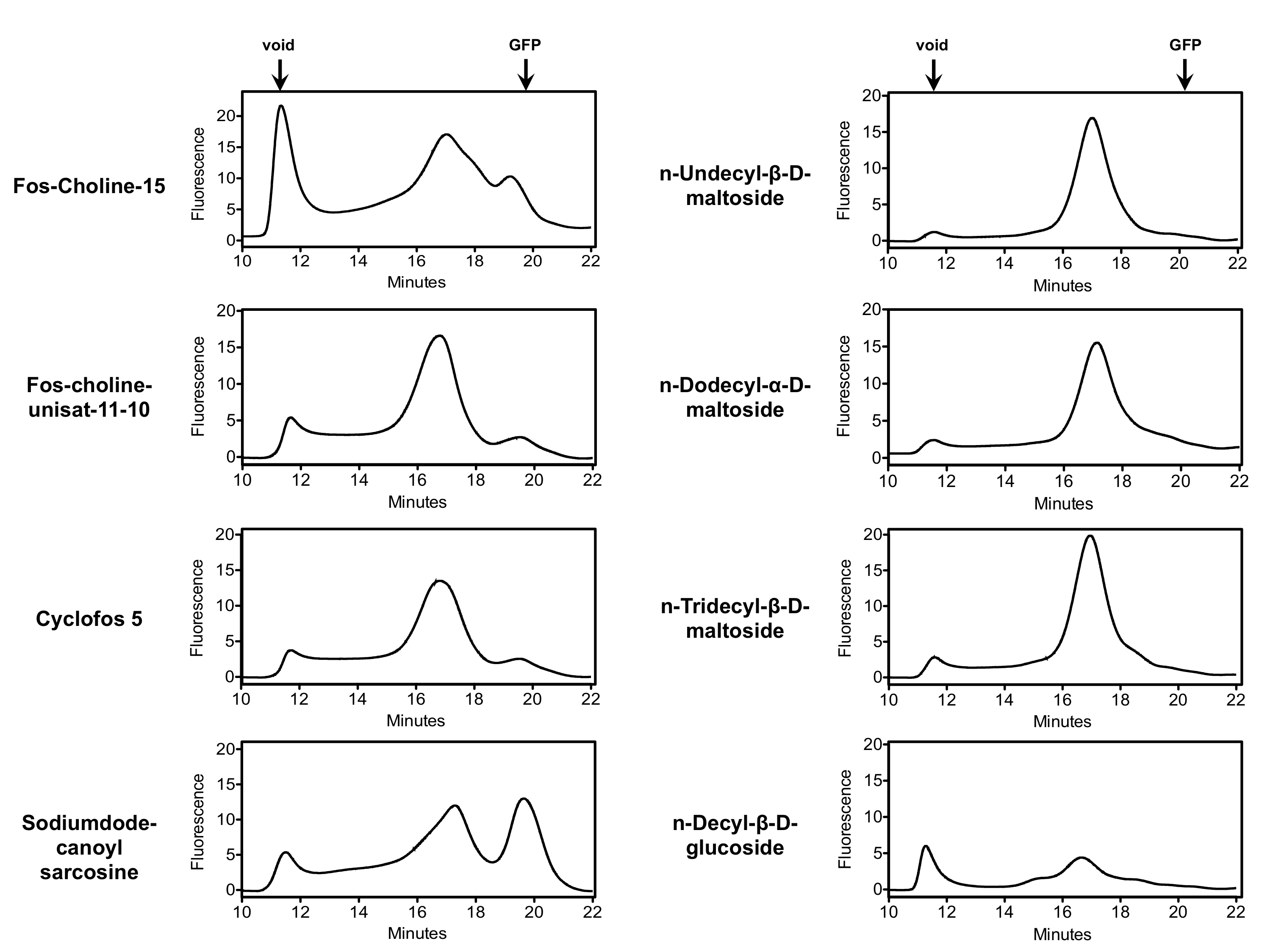


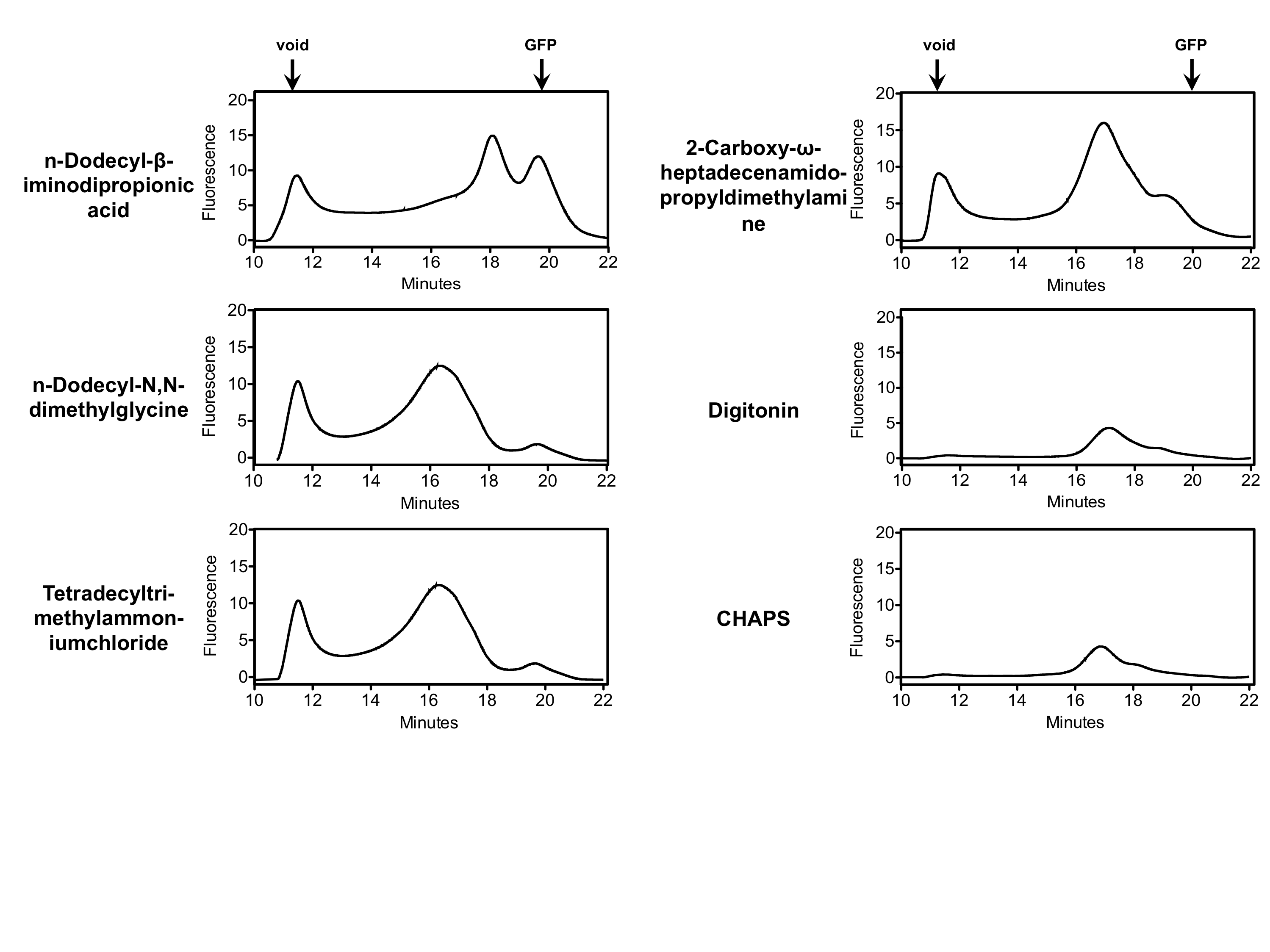


**Supplementary Table S1**

| **Position** | **Detergent** | **cmc %** | **used %** | **nature** |
| --- | --- | --- | --- | --- |
| **A1** | Anameg®-7 | 0.65 | 1% | N |
| **A2** | Anapoe®-20 | 0.0072 | 1% | N |
| **A3** | Anapoe®-35 | 0.001 | 1% | N |
| **A4** | Anapoe®-58 | 0.00045 | 1% | N |
| **A5** | Anapoe®-80 | 0.0016 | 1% | N |
| **B1** | Anapoe®-C10E6 | 0.025 | 1% | N |
| **B2** | Anapoe®-C10E9 | 0.053 | 1% | N |
| **B3** | Anapoe®-C12E8 | 0.0048 | 1% | N |
| **B4** | Anapoe®-C12E9 | 0.003 | 1% | N |
| **B5** | Anapoe®-C12E10 | 0.2 | 1% | N |
| **C1** | Anapoe®-C13E8 | 0.0055 | 1% | N |
| **C2** | Anapoe®-X-100 | 0.015 | 1% | N |
| **C3** | Anapoe®-X-114 | 0.011 | 1% | N |
| **C4** | Anapoe®-X-305 | ­– | 1% | N |
| **C5** | Anapoe®-X-405 | 0.16 | 1% | N |
| **D1** | Big CHAP | 0.25 | 1% | N |
| **D2** | Big CHAP deoxy | 0.12 | 1% | N |
| **D3** | CYGLU®-3 | 0.86 | 2% | N |
| **D4** | CYMAL®-1 | 15 | 2% | N |
| **D5** | CYMAL®-2 | 5.4 | 2% | N |
| **E1** | CYMAL®-3 | 0.37 | 1% | N |
| **E2** | 2.6-Dimethyl-4-heptyl-β-D-maltose | 1.2 | 2% | N |
| **E3** | 2-propyl-1-pentyl maltose | 1.9 | 2% | N |
| **E4** | MEGA-8 | 2.5 | 2% | N |
| **E5** | n-Octyl-β-D-glucoside | 0.53 | 2% | N |
| **F1** | n-Nonyl-β-D-glucoside | 0.2 | 1% | N |
| **F2** | n-Octyl-β-D-maltoside | 0.89 | 2% | N |
| **F3** | n-Nonyl-β-D-maltoside | 0.28 | 2% | N |
| **F4** | n-Decyl-α-D-maltoside | – | 1% | N |
| **F5** | n-Tetradecyl-β-D-maltoside | 0.00054 | 1% | N |
| **G1** | n-Undecyl-α-D-maltoside | 0.029 | 1% | N |
| **G2** | n-Undecyl-β-D-maltoside | 0.029 | 1% | N |
| **G3** | n-Dodecyl-α-D-maltoside | 0.0076 | 1% | N |
| **G4** | n-Dodecyl-β-D-maltoside | 0.0087 | 1% | N |
| **G5** | n-Tridecyl-β-D-maltoside | 0.0017 | 1% | N |
| **H1** | n-Heptyl-β-D-thioglucoside | 0.85 | 2% | N |
| **H2** | n-Octyl-β-D-thiomaltoside | 0.4 | 2% | N |
| **H3** | n-Nonyl-β-D-thiomaltoside | 0.15 | 1% | N |
| **H4** | n-Decyl-β-D-thiomaltoside | 0.045 | 1% | N |
| **H5** | n-Undecyl-β-D-thiomaltoside | 0.011 | 1% | N |
| **I1** | n-Dodecyl-β-D-thiomaltoside | 0.0026 | 1% | N |
| **I2** | Pentaethylene glycol monodecylether(C10E5) | 0.031 | 1% | N |
| **I3** | Tetraethylene glycol monoethylether(C8E4) | 0.25 | 1% | N |
| **I4** | Sucrose monododecanoate | 0.016 | 1% | N |
| **I5** | Dimethyldecylphosphine oxide | 0.1 | 1% | N |
| **J1** | Tripglu | 3.6 | 2% | N |
| **J2** |  |  |  |  |
| **J3** | Decyl-β-D-glucoside | 0.07 | 1% | N |
| **J4** |  |  |  |  |
| **J5** | CYGLU®-4 | 0.058 | 2% | N |
| **K1** | CYMAL®-5 | 0.12 | 2% | N |
| **K2** | MEGA-10 | 0.21 | 2% | N |
| **K3** | NP40 | 0.05-0.3 | 1% | N |
| **K4** | Cyclohexyl-n-hexyl-β-D-maltoside | – | 1% | N |
|  |  |  |  |  |
| **C6** | Anzergent® 3-10 | 1.2 | 2% | Z |
| **C7** | Anzergent®3-12 | 0.094 | 1% | Z |
| **C8** | Anzergent® 3-14 | 0.007 | 1% | Z |
| **C9** | CHAPS | 0.49 | 2% | Z |
| **C10** | CHAPSO | 0.5 | 2% | Z |
| **D6** | C-DODECAFOS™ | 0.77 | 2% | Z |
| **D7** | Cyclofos™-4 | 0.45 | 2% | Z |
| **D8** | Cyclofos™-5 | 0.15 | 1% | Z |
| **D9** | Cyclofos™-6 | 0.094 | 1% | Z |
| **D10** | Cyclofos™-7 | 0.022 | 1% | Z |
| **E6** | Cyclofos™-2 | 7.5 | 1% | Z |
| **E7** | Fos-Choline®-9 | 1.2 | 2% | Z |
| **E8** | Cyclofos™-3 | 1.3 | 2% | Z |
| **E9** | Fos-Choline®-11 | 0.062 | 1% | Z |
| **E10** | Fos-Choline®-12 | 0.047 | 1% | Z |
| **F6** | Fos-Choline®-13 | 0.027 | 1% | Z |
| **F7** | Fos-Choline®-14 | 0.0046 | 1% | Z |
| **F8** | Fos-Choline®-15 | 0.0027 | 1% | Z |
| **F9** | Fos-Choline®-16 | 0.00053 | 1% | Z |
| **F10** | Fos-Choline®-Iso-9 | 0.99 | 2% | Z |
| **G6** | Fos-Choline®-Iso-11 | 0.9 | 2% | Z |
| **G7** | Fos-Choline®-Iso-11-6U | 0.87 | 2% | Z |
| **G8** | Fos-Choline®-Unisat-11-10 | 0.21 | 1% | Z |
| **G9** | Fos-Choline®-8 | 3.4 | 2% | Z |
| **G10** | Fosfen™-9 | 0.014 | 1% | Z |
| **H6** | Nopol-Fos™ | 1.4 | 2% | Z |
| **H7** | PMAL™-8 | – | 1% | Z |
| **H8** | PMAL™-C10 | – | 1% | Z |
| **H9** | n-Decyl-N.N-dimethylglycine | 0.46 | 2% | Z |
| **H10** | n-Dodecyl-N.N-dimethylglycine | 0.041 | 1% | Z |
|  |  |  |  |  |
| **I7** | n-Tetradecyl-N.N-dimethylamine-N-oxide | 0.0075 | 1% | Z |
| **I8** | n-Dodecyl-N.N-dimethylamine-N-oxide | 0.023 | 1% | Z |
| **I9** | Tripao | 4.5 | 2% | Z |
| **I10** | n-Tetradecyl-N.N-dimethylamine-N-oxide | 0.0075 | 1% | Z |
| **J6** | LAPAO | 0.052 | 2% | Z |
| **J7** | PMAL™-C-12 | ­– | 2% | Z |
| **J8** | 2-Carboxy-w-heptadecenamidopropyldimethylamine | – | 1% | Z |
| **J9** | 2-Carboxy-5-pentadecenamidopropyldimethylamine | – | 1% | Z |
| **J10** | N.N dimethyl(3-carboxy-4-dodec-5-ene)aminopropylamine | 0.0178 | 1% | Z |
|  |  |  |  |  |
| **A6** | Deoxycholic acid. sodium salt | 0.24 | 1% | A |
| **A7** | Sodium cholate | 0.41 | 2% | A |
| **A8** | Fosmea®-10 | 0.15 | 1% | A |
| **A9** | Sodium dodecanoyl sarcosine | 0.42 | 2% | A |
| **A10** | n-Dodecyl-β-iminodipropionic acid (only used for MDR3) | N/A | 1% | A |
|  |  |  |  |  |
| **B6** | Decyltrimethylammonium chloride | 0.07 | 1% | C |
| **B7** | Dodecyltrimethylammonium chloride | 0.0012 | 1% | C |
| **B8** | Hexadecyltrimethylammonium chloride | 0.000102 | 1% | C |
| **B9** | Tetradecyltrimethylammonium chloride | 0.0009 | 1% | C |
